# An automated, low-latency environment for studying the neural basis of behavior in freely moving rats

**DOI:** 10.1186/s12915-023-01660-9

**Published:** 2023-08-11

**Authors:** Maciej M. Jankowski, Ana Polterovich, Alex Kazakov, Johannes Niediek, Israel Nelken

**Affiliations:** 1 The Edmond and Lily Safra Center for Brain Sciences and the Department of Neurobiology, Silberman Institute of Life Sciences, the Hebrew University of Jerusalem, Jerusalem, Israel; 2 BioTechMed Center, Multimedia Systems Department, Faculty of Electronics, Telecommunications and Informatics, Gdansk University of Technology, Gdansk, Poland

**Keywords:** Complex behavior, Automated environment, Brain, Insular cortex, Auditory cortex, Electrophysiology, Freely moving rat

## Abstract

**Background:**

Behavior consists of the interaction between an organism and its environment, and is controlled by the brain. Brain activity varies at sub-second time scales, but behavioral measures are usually coarse (often consisting of only binary trial outcomes).

**Results:**

To overcome this mismatch, we developed the Rat Interactive Foraging Facility (RIFF): a programmable interactive arena for freely moving rats with multiple feeding areas, multiple sound sources, high-resolution behavioral tracking, and simultaneous electrophysiological recordings. The paper provides detailed information about the construction of the RIFF and the software used to control it. To illustrate the flexibility of the RIFF, we describe two complex tasks implemented in the RIFF, a foraging task and a sound localization task. Rats quickly learned to obtain rewards in both tasks. Neurons in the auditory cortex as well as neurons in the auditory field in the posterior insula had sound-driven activity during behavior. Remarkably, neurons in both structures also showed sensitivity to non-auditory parameters such as location in the arena and head-to-body angle.

**Conclusions:**

The RIFF provides insights into the cognitive capabilities and learning mechanisms of rats and opens the way to a better understanding of how brains control behavior. The ability to do so depends crucially on the combination of wireless electrophysiology and detailed behavioral documentation available in the RIFF.

**Supplementary Information:**

The online version contains supplementary material available at 10.1186/s12915-023-01660-9.

## Background

Behavior involves brain-controlled, complex interactions between an organism and its environment. Natural behavior has many components. Much of what we understand about brains and their functions was shaped by examinations of single components of the interaction between organisms and their environments, including sensation and perception [1]; motor control [2]; decision making [3]; and memory [4]. However, these successes have been achieved at a price—animals are often studied using behavioral tasks that are much simpler than those they face in their natural habitats.

The main observable output of behavior is movement—animals select and time their actions. Patterns of movements may be rich: predictive movements may precede trial events when there is a temporal structure to the task; movements may differ between individuals; and movements may be used for solving cognitive tasks. For example, rats exert active control on selected motor variables in order to enable consistent perception of location when using their whiskers [5].

Evidently, even in simple tasks, animals perform much more than a single action at each trial. In complex, naturalistic settings, the amount of movement (and number of decisions) performed by animals is substantial. Brain activity is expected to reflect and shape the full complexity of the concomitant behavioral strategies [6]. During a trial, neural activity throughout the brain runs its fast course, before, during, and after relevant decision points. For example, movements that vary from trial to trial account for much of the inter-trial variability in wide-field calcium imaging of mouse cortex [6].

We designed and constructed the Rat Interactive Foraging Facility (RIFF) to allow high-resolution behavioral data acquisition in time and space combined with tetherless electrophysiology in freely moving animals. The RIFF is controlled by a real-time loop that tracks the rat, identifies its actions, and reacts to them. Because of the generic nature of this loop, it is flexible and can implement a rich set of behavioral scenarios.

We describe here the design principles of the RIFF and provide detailed information about its implementation. The control program and all essential post-processing programs are described and made available in the associated repository, together with exemplary data. We illustrate the use of the RIFF with two very different tasks. Rats learned these behavioral tasks efficiently, sometimes within a few hours. Different rats developed individual patterns of behavior, which can be detected early in the learning process and tracked over months. We show that neural activity recorded in primary and higher-order auditory fields tracks relevant behavioral parameters such as animal location and pose in addition to responding to auditory stimuli.

## Results

### Overview of the RIFF

The Rat Interactive Foraging Facility (RIFF) is designed to jointly study behavior and brain activity of freely moving animals interacting with a rich environment that is nevertheless amenable to good experimental control.

**Fig. 1:**
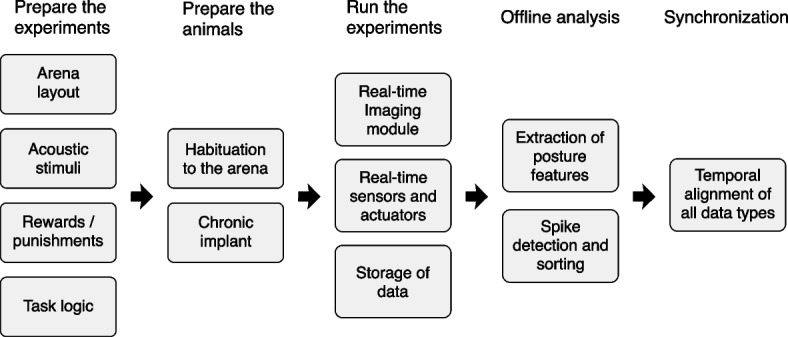
Workflow for the design, preparation, execution, and initial analysis of behavioral experiments as they pertain to the RIFF. The design of an auditory-guided task includes the development of task logic, the nature of rewards and punishments, as well as the selection of the auditory stimuli. The flexibility of the RIFF allows for substantial freedom in these choices. Rat preparation includes habituation to the RIFF and electrode implantation. While running the experiment, the RIFF is designed to keep a tight synchronization of all real-time events, and to ensure efficient data storage. The stored data allows for offline analysis that includes spike sorting and animal pose estimation, which are then precisely aligned with the rest of the information from the experiment

The implementation of the RIFF requires integration of a large number of interacting systems and processes (Fig. 1), which we kept as separate and modular as possible in order to allow for modifications and extensions as experience is gained and new technology becomes available. While the logic used to operate the RIFF can be very flexible, we intended it to operate mostly as a Markov Decision Process (MDP) [7]. MDPs are “state machines.” They are defined by a set of states (defined, for example, by the location of the animal, the current stimulus that is presented, and the correct ports to poke in order to get a reward), a set of actions that an animal can take in each state, and a set of actions that can be taken by the environment (typically providing rewards and punishments to the animal). The MDP is governed by two (potentially stochastic) rules. The first is the rule by which one state follows another. In an MDP, transitions depend only on the current state of the process and on the current action of the animal. The second rule prescribes whether and which actions the environment takes, depending only on animal action and the consequent state transition.

**Fig. 2:**
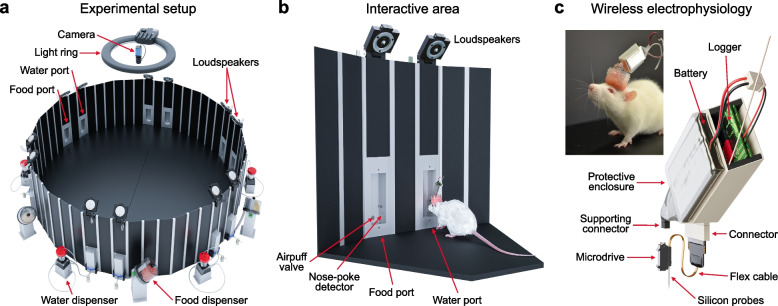
**a** An experimental arena for studying the neural basis of auditory-guided complex behavior. The RIFF is a large arena (160 cm in diameter), equipped with 12 loudspeakers, 6 food dispensers and 6 fluid dispensers, 12 airpuff outlets, and a ceiling mount camera. The wall panels can be removed, the ports can be closed by blinds, and the spatial arrangement of the environment can be adjusted for different experiments. **b** Close-up on one interaction area. An adult female rat with a chronic implant and a neural logger is shown for scale. Each interaction area consists of two ports, one for food rewards and the other for fluids. Each port has a nose-poke detector and airpuff outlet. Two speakers are mounted above each port. **c** An approach for chronic wireless recordings in rats. The neural logger and battery are in the protective plastic case. A multi-contact silicon probe is mounted on the microdrive. The device is inclined to the back to allow rats to move naturally with an undisturbed access to the ports of the interaction area. The picture shows a rat with a 32-channel moveable silicon probe implant and wireless data logger. The battery used here allows for 3 h of recordings

The behavioral environment is a large 18-sided polygon (Fig. 2a) with six interaction areas (IAs), each consisting of two loudspeakers, a water port, a food port, and airpuff valves (Fig. 2b). The main sensor for animal location is a ceiling-mounted camera with a dedicated computer that performs real-time image processing. The camera tracks the location of the rat with a temporal resolution of 30 ms, and the location information is transmitted to the main computer. Additional sensors include the nose-poke detectors at each feeding port that report their state in real time; the status of the food and water ports; and analog sensors carried by the animals such as a microphone, accelerometers, and gyroscopes [8]. The RIFF interacts with the freely moving animal through the actuators (Fig. 2b). In the present implementation, the response repertoire of the RIFF includes food or water delivery, airpuff delivery, and sound presentation by any combination of the 12 speakers. Neural responses are recorded using chronically implanted moveable silicon probes (Fig. 2c). More details about the design of the RIFF can be found in Additional files 1 (an overview of the RIFF and its control systems) and 2 (dimensions of the most important components of the arena).

**Fig. 3:**
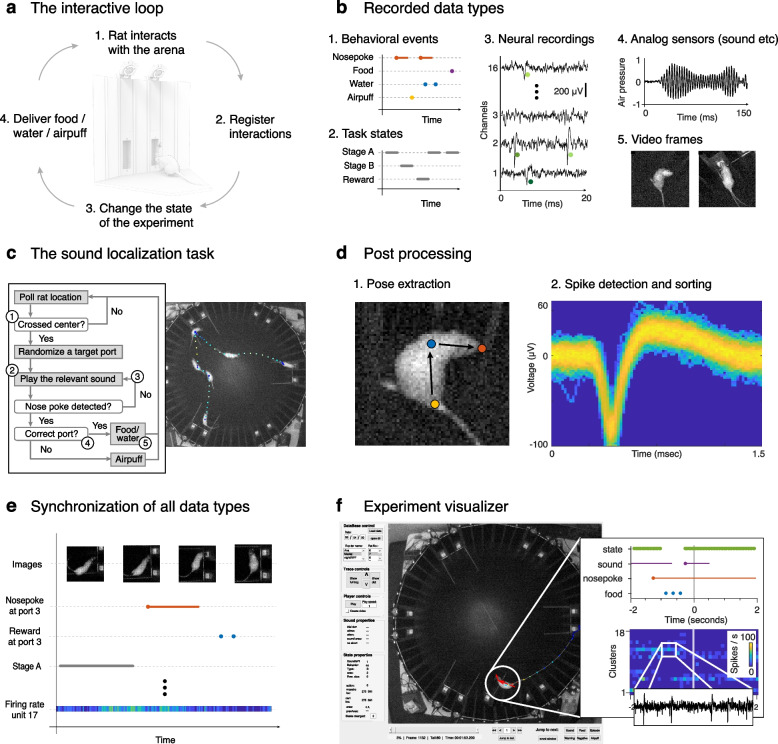
Operation of the RIFF. **a** Rats interact with the RIFF through movement and nose pokes. Their location is identified online using the video camera and a dedicated computer. The experimental environment reacts to rat actions by changing its state and providing different types of feedback—sounds, rewards, or punishments. **b** The RIFF collects multiple data types: behavioral events (e.g., nose pokes, rewards, punishments); task states; neuronal activity; analog sensor signals (microphone, motion sensors); and the animal location tracked by the ceiling mount camera.** c** An illustration of a sequence of interactions between a rat and the RIFF during the LD task. (1) The rat moved to the center of the arena in order to initialize the trial. (2) Once it crossed into the central area (a circle with a radius of 30 cm), the RIFF started sound presentation from both loudspeakers in a randomly selected interaction area. (3) Initially, in the example presented here, the rat approached a wrong port; a second sound presentation caused it to move towards the correct target port (4), and to receive the reward (5). **d** Data post-processing. Posture features are extracted using a custom-trained DNN. The Kilosort2 program used with a custom wrapper performs largely automatic spike detection and sorting. **e** All data types are synchronized on a single time axis and **f** can be visualized offline using a custom visualizer software. The visualizer can be used to browse through all synchronized data types up to the level of the raw neural signals

The logic of the RIFF is implemented in a Matlab program that runs on the main control computer (Fig. 3a; green in Additional file 1), collecting the data from sensing hardware (Fig. 3b), and driving the actuators using a predefined logic (an example is described in Fig. 3c; see also “Methods” and Additional file 3). Five major data types are recorded during the experiment and stored for offline analysis (Fig. 3b): time stamps of behavioral events; time stamps of internal state transitions; neural activity; analog sensor data; and video images.

We implemented a post-processing data extraction and analysis pipeline that is almost completely automated. A convolutional neural network estimates the locations of the nose, neck, and the base of the tail of the rat from the video stream, inferring head and body directions (Fig. 3d (1), Additional files 4, 5 and 6). The neural recordings are denoised, and spike detection and sorting is performed by a custom wrapper to Kilosort2 [9, 10] (Fig. 3d (2), Additional file 7). After extracting information from each data stream, all data are synchronized to a single time axis (Fig. 3e). The output is a single file that combines all data types and additional extracted features. The post-processing steps are much faster than actual experiment time. An interactive graphical interface (Fig. 3f, Additional file 8) is used to verify the experiment logic and data integrity, and to examine the fine details of the behavior and the data.

Further details on the design and performance of the RIFF are provided in the “Methods” section and in the software repository and its wiki section at [11]. The software repository contains the main control loop for the two tasks described below, and the wiki has a guided example showing how to modify it for implementing different tasks. The repository also includes the full post-processing pipeline and the visualizer together with two data sets that can be downloaded, processed through the offline processing pipeline, and displayed on the visualizer.

An important feature of the RIFF is the tight synchronization between behavior and electrophysiology. There are two facets to this synchronization. First, every device (video camera, port sensors and actuators, and the sound card) is synchronized to the same time base by TTL pulses that are co-registered together with the electrophysiological data (Additional file 1, blue arrows indicate these timing signals). Thus, the neural activity can be synchronized to any event at the precision at which the hardware systems register these events. In consequence, sound onsets are synchronized with the electrophysiological activity to the sampling precision of the electrophysiological data (30 or 40 kHz, depending on the data collection system). On the other hand, the synchronization of the video frames is limited by the much slower frame rate of the camera.

Second, we aimed to achieve a short latency between behavioral events and the consequent response of the RIFF equipment. The most important delay for both tasks is the time from the moment a video frame is taken to the moment in which the main loop of the RIFF is notified about the position of the rat and can react to it. This latency was 39.5 ± 11.8 ms, with a unimodal, bounded distribution (mean ± std, min=11.3 ms, max=61.5 ms, *n*=186). A complete description of the latencies in the RIFF is provided in the “Methods” section.

### Rats rapidly learned a complex task

To illustrate the flexibility of the RIFF, we implemented two different behavioral tasks: a multiple-strategy (St+) task and a localization/discrimination (L/D) task. Both tasks and their rationale are described in detail in the “Methods” section. For details and illustrative movies, see Additional files 3, 9 and 10.

The full St+ task has a number of components, of which we illustrate here only the central one—the set of predefined rules and contingencies that enable rats to adopt their own preferred policy for reward optimization in a complex appetitive situation. This was done in preparation for recordings in the insular auditory field, where hunger and satiety are expected to have an effect on the auditory responses. Furthermore, the full task included passive sessions that made it possible to compare neural activity before, during, and after the active session.

The L/D task is a standard auditory task. Here we describe the main condition, in which rats had to identify the location of the reward interaction area (IA) using both sound localization and sound identity cues. Sound localization cues were supplied by playing the sound from the loudspeakers of the reward IA; identity cues were supplied by using different sounds for each IA. Like the St+, the full task included additional conditions, in which rats had to identify the rewarded IA using only location cues (using the same sound at all IAs) or using only sound identity cue (by playing the sound for the specific IA from all 12 loudspeakers).

Here we use the two tasks only as an illustration for the flexibility of the RIFF. Both tasks were run daily in the same setup with different subjects.

We illustrate rapid learning in the RIFF using the St+ task. Each behavioral episode of the St+ task started with an attention sound, followed by a period of 2.5 s during which the rat could control the selection of the IA for the next reward by moving to appropriate positions in the arena. Next, this reward location was communicated to the rat by a target sound emitted by the loudspeakers of the selected IA. The rat had to poke in one of the two ports of the target IA within 20 s of the presentation of the target sound. A poke or a timeout resulted in a feedback sound followed by an inter-trial interval (3 s) until the next attention event. Rats rapidly increased the amount of reward they received while performing the task despite its non-trivial structure (see Additional file 9). We analyze here in detail the first 2 days of task exposure.

**Fig. 4:**
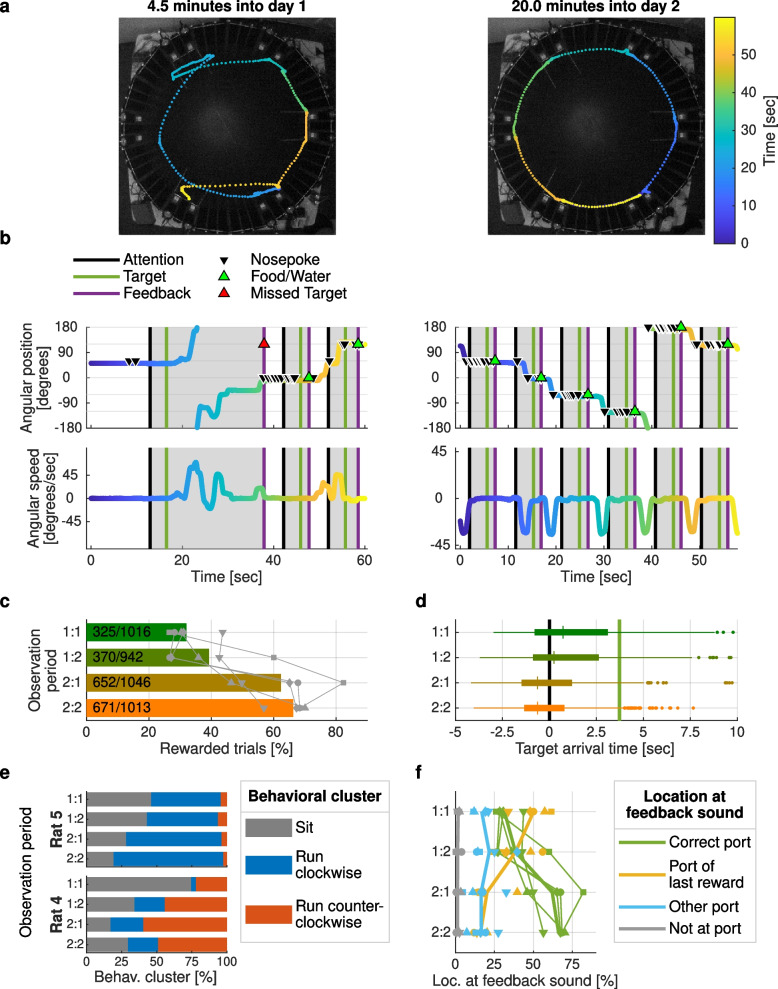
Rapid learning in the RIFF. **a** One-minute trajectory segments from 70-min training sessions on days 1 and 2 of learning. **b** Angular position and speed for the same trajectories. Trials (from attention to feedback) are marked in gray. Nose pokes, rewards, and missed targets are indicated by triangles. By day 2, rats have developed a stereotypic running pattern. **c** Success rate improved over time. Observation periods 1:1/1:2 denote the first/second half of the session on day 1; 2:1/2:2 denote session halves on day 2. Bars indicate average success rates from five rats; gray symbols mark individual rats. **d** Rats learned the temporal structure of the task. Shown are arrival times relative to the attention sound (black line; target sound, green line), calculated from all trials of five rats. **e** Different rats learned different strategies. Each trial was classified as either sitting, clockwise running, or counterclockwise running. Rat 4 preferred counterclockwise running, while Rat 5 avoided counterclockwise running. **f** Learning to move was the most important contribution to performance improvement. Each trial was classified as either “correct port,” “port of last reward,” “other port,” or “not at port.” Average proportions of each location across five rats are displayed as thick lines, individual rats are displayed as symbols and thin lines. The proportion of “correct” locations increased from one observation period to the next, and the proportion of “port of last reward” locations decreased over time

Each rat trained in the RIFF for 70 min per day. During the first 2 days of training, rats quickly learned to move from one IA to the next. Figure 4a shows 1-min segments of a rat’s trajectory on day 1 and day 2. Erratic loops and hesitations were present on day 1 but disappeared on day 2. Figure 4b shows the angular position and angular speed from the same trajectories, along with a schematic illustration of the trial structure. Clearly, the stereotypic running pattern of day 2 was still absent at the beginning of day 1.

To analyze changes in behavior within and across sessions, we subdivided each session into two halves, denoting the four resulting observation periods by 1:1, 1:2, 2:1, and 2:2. The percentage of rewarded trials—a simple measure of learning—increased from 32% in observation period 1:1 to 66% in observation period 2:2 (Fig. 4c). We modeled the percentage of rewarded trials as a function of the observation period using a linear mixed effects (LME) model with random intercepts for rats. The model had a significant main effect of observation period with a slope corresponding to an average improvement of 13% ± 2% (mean ± STE) additional rewarded trials from one observation period to the next (*t*(18) = 5.95; *p* = 1.2×10^−5^). As rats learned to position themselves to collect rewards, they adapted to the timing of the task: rats arrived in the IA of an upcoming reward 1 s earlier on day 2 compared to day 1 (Fig. 4d; median arrival time for successful trials on day 1, 0.4 s (interquartile range (IQR) 3.7 s) after attention sound; on day 2, 0.6 s (IQR 2.4 s) before attention sound). This suggests that rats learned to correctly predict the upcoming reward time. We modeled the logarithm of arrival times as an LME with the observation period as a fixed factor and with random intercepts for rats (times were shifted to start 5.5 s before the attention sound to turn all arrival times into positive numbers; the logarithmic transformation was selected in order to reduce the skewness of the distribution of arrival times). There was a significant effect of observation period (estimate −0.069 ± 0.008; *t*(1829) = −8.86; *p* = 1.8×10^−18^), so that the arrival time shifted to earlier times by a factor of exp(−0.069 ± 0.008) = 0.93 ± 0.007 from one observation period to the next. A comparison of the distributions of arrival times on day 1 and day 2, in each rat is shown in Additional file 11. Arrival before the attention sound was more likely on day 2 than on day 1 (red bars preceding black line in Additional file 11), and arrival after the target sound was less likely on day 2 than on day 1 (blue bars following green line in Additional file 11), showing that rats learned to time their actions according to the task structure and to predict the timing of the upcoming attention sound. Arrival time distributions significantly differed in each rat (two-sample Kolmogorov-Smirnov test; *p* < 0.01 in each rat; *p* = 4.62 × 10^−15^ for all rats), and arrival times on day 2 were significantly earlier in four out of the five rats (two-sample *t*-test; *p* < 0.006 in rats 4, 6, 7, 8; *p* = 0.13 in rat 5; *p* = 1.13 × 10^−26^ for all rats).

The velocity trajectories in Fig. 4a suggest that the movement patterns of the rats quickly became stereotypical. We calculated the maximal angular speed in both the clockwise and counterclockwise directions during each trial (analysis time window, 0.5 to 9 s after the feedback sound of the previous trial). All rats used three clearly distinct behavioral motives: “not moving,” “clockwise running,” and “counterclockwise running” (Additional file 12 for cluster analysis). Each experimental session can thus be described as a sequence of these three motives, timed by the rat to harvest rewards from the RIFF.

We analyzed how the distribution of the three motives evolved during the four observation periods. There was a large decrease in the number of trials in which the rat did not move. We modeled the proportion of “not moving” trials as a linear function of the observation period using an LME with random intercepts for rats. Observation period had a significant main effect (estimate, −0.14 ± 0.02; *t*(18) = −5.89; *p* = 1.4×10^−5^). Thus, on average, the proportion of “not moving” trials decreased by 14% ± 2% from one observation period to the next: rats learned to move more often as training progressed.

Importantly, rats showed a clear, individual preference for running in one direction or the other. Thus, we found that the proportion of “clockwise running” out of all running trials depended on the rat (two rats shown in Fig 4e; *χ*^2^-test for independence; *χ*^2^ (24) > 701 for the five rats together; *p* < 10^−20^). In both rats, the fraction of “not moving” trials (shown in gray in Fig. 4e) generally decreased over time. Rat 4 preferentially ran counterclockwise (blue), while rat 5 preferentially ran clockwise (red). The distribution of “clockwise”/“counterclockwise” significantly depended on rat identity (these two rats: *χ*^2^(21) > 77.6, *p* < 10^−20^ in each observation period).

Lastly, to better understand the nature of failed trials, we classified the rat location at the time of the feedback sound into four types: “correct port” (rewarded trials), “port of last reward,” “another port,” and “not at any port.” As training progressed, rats reached the correct port more often (green line in Fig. 4f). The most common error type consisted of remaining in a reward location after a reward, thereby missing the next reward opportunity which was always at a different port (orange line in Fig. 4f). This type of error decreased substantially from day 1 to day 2. Statistical analysis confirmed that the distribution of rat location types depended on the observation period (*χ*^2^ (245) = 617, *p* < 10^−20^ for all rats; *χ*^2^ (29) > 38.4, *p* < 1.5×10^−5^ in each individual rat). To quantify the interaction between the location types and observation periods, we modeled the probability of the location types as an LME model in which the probability for each location type was a linear function of the observation period with a slope that depended on the location type, with random intercepts for each location type in each rat. This model revealed a significant interaction between observation period and location type (F(3,72) = 44.1; *p* = 2.8×10^−16^), confirming that over time, the rats changed their preferred location types. Indeed, the slope of the “correct port” location type was 0.13 ± 0.02 (*t*(72) = 8.36; *p* = 3.2×10^−12^), confirming the increase in correct trials over time. In contrast, the slope for “port of last reward” location type was −0.12 ± 0.02, showing a decrease in this type of error. The difference between these two slopes was highly significant (*t*(72) = −11.5; *p* = 6.1×10^−18^).

### Neural correlates of behaviors

Rats in the St+ task were implanted with electrodes targeting the auditory field in the posterior insular cortex (Ins), which in rats is anatomically separate from other auditory cortical fields [12, 13]. Rats in the L/D task were implanted with electrodes which traversed the primary auditory field (AC) at least in part of their trajectory.

**Fig. 5:**
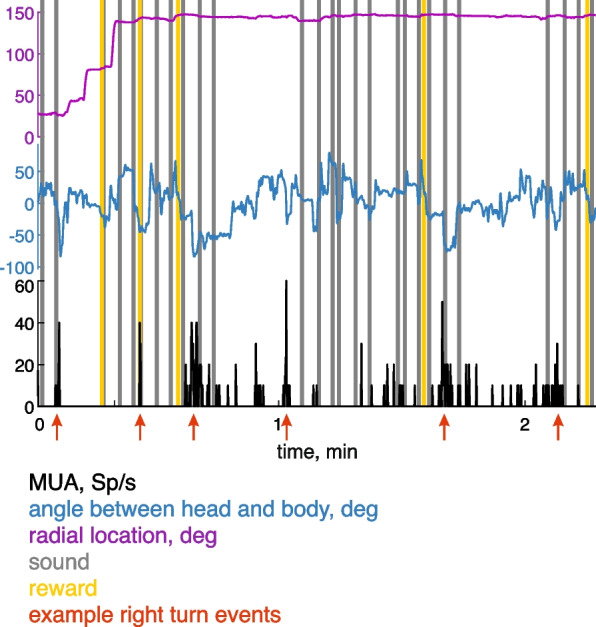
Example of concurrent electrophysiology and behavioral measurements. The neuronal activity of a unit in Ins is shown at the bottom (black), together with a plot of the angle between head and body (blue, middle) and radial location in the arena (magenta, top). The trace shows the activity over 2 min of recording, starting at 15 min after session beginning. This is the same unit whose responses are analyzed in Fig. 6k. Red arrows at the bottom indicate head turns to the right, which are accompanied by an increase in firing rate. Gray lines indicate sound presentation onsets and yellow lines are reward times. Note that the activity of the unit does not seem to be related to radial location, sound times, and rewards

**Fig. 6:**
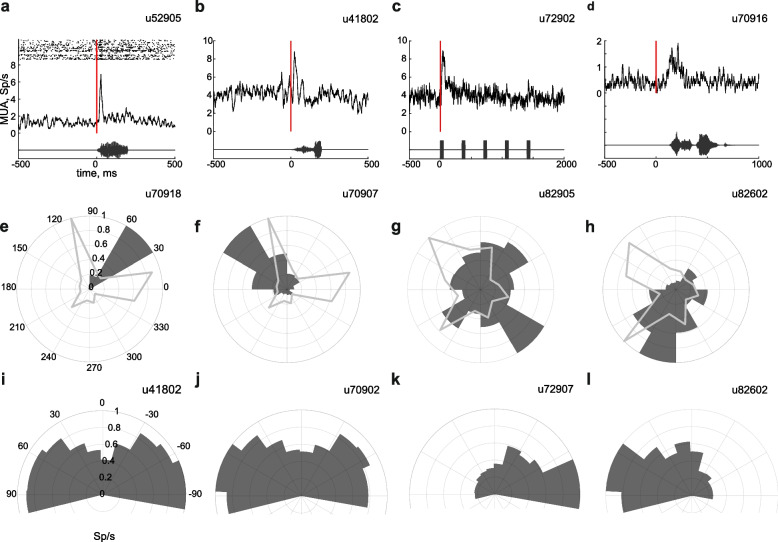
Neural responses during behavior. (**a**-**d**) Sound driven responses. Mean over one session of the sound driven responses (top) and the oscillograms of the corresponding auditory stimuli (bottom). Red line indicates sound onset. The dots in (**a**) show the spike times in the individual trials as a raster display. (**e**-**h**) Location sensitive units. Each panel shows the mean firing rate of a unit while the animal was in one of 12 sectors of the arena (dark gray), and the time spent in each sector (light gray). (**i**-**l**) Units sensitive to angle between head and body of the animal. The normalized mean firing rate is plotted when the rat exhibited different angles between head and body (dark gray). The last bin on each side includes the data equal and more extreme than the indicated angles. Unit identifiers are marked above each panel

In addition to the behavioral data, the RIFF pipeline produced traces of firing rates of the sorted units. Behavior and firing rates are synchronized with a precision of a few milliseconds. Figure 5 shows an example. These are the synchronized traces of the firing rate of one unit in Ins, together with the angle between head and body and the angular location of the rat in the RIFF. Stimulus presentation onsets and reward times are presented as well. In this trace, the highly phasic firing pattern of the unit seem to be sensitive to the angle between the head and the body of the animal: when the animal looked to the right (negative angles), there were often bursts of firing (red arrows in Fig. 5). The unit does not seem to be responding to angular location, to sounds, or to rewards (see Fig. 6k for a summary and a statistical analysis of the dependence of the responses of this unit on head-to-body angle).

Units in both the AC and Ins showed responses to sounds. Figure 6a and b show responses of units in AC of two rats. The stimuli were word-like stimuli (200 ms word excerpts, with their spectral envelopes shifted and stretched to fit a 1–40 kHz range; see “Methods”). These stimuli were some of the target sounds in the L/D task (mean response of *N* = 96 and *N* = 143 sound presentations in Fig. 6a and b respectively; here and elsewhere in this paragraph a paired *t*-test was performed between spike counts in the response window and in a window of the same duration just preceding stimulus onset, *t*(190)=3.26, *p* = 0.0013 and *t*(284)=2.63, *p* = 0.0090 respectively). Both units had short-latency (<20 ms) onset responses. Figure 6c and d show responses of units in Ins. The stimulus in Fig. 6c was the attention sound (a train of short broadband periodic sound bursts with a pitch of 2 kHz). The unit responded to the onset of the first sound burst in the train (mean of *N* = 233 sound presentations, *t*(464) = 4.48, *p* = 9.6×10^−6^). Figure 6d shows a unit with a response that was loosely locked to a word-like stimulus (mean of *N* = 29 sound presentations, *t*(56) = 3.27, *p* = 0.0018).

Remarkably, in both areas, we found units whose activity was strongly modulated by non-auditory aspects of the task and the state of the rats. Two of these classes of responses are illustrated here.

In Ins, we found units that increased their firing rates selectively at specific locations in the arena (Fig. 6e–h). Since the rats spent most of their time close to the walls of the RIFF in the St+ task, the data are summarized as polar plots. For example, the unit shown in Fig. 6e responded essentially only when the rat was at one specific sector (polar pie chart; 1-way ANOVA, F(11,35050) = 908, *p* = 0). This sector was not preferred by the rat: it spent much more time in the two flanking sectors (line plot in Fig. 6e). Units in Fig. 6f–h also responded significantly stronger for one of the sectors (1-way ANOVA for each unit separately: F(11,305050) = 463, *p* = 0; F(11,41663) = 5.89, *p* = 1.21×10^−9^; F(11,34830) = 91.1, *p* = 9.10×10^−205^, respectively). To study the spatial resolution of the firing rates at a higher resolution, Additional file 13 shows the same data at a resolution of 1°. The firing rate of the location-sensitive units sometimes changed twofold or more within 5° of the arena (see Additional file 13), corresponding to an arc length of about 7 cm near the wall of the arena. With a running speed of up to 80 cm/s, the rats were able to go through such a section within less than 100 ms. Thus, our ability to document these apparent spatially sensitive units depended crucially on high temporal resolution and tight synchronization between the video stream and the electrophysiological data.

To control for potential confounds, we also tested the significance of the relationships between firing rates and location using a permutation test (see “Methods”). The four units displayed here passed this test with *p*<0.01. We then checked that the firing rate of these units was not determined by the time spent in each sector and that their location sensitivity was largely independent of their sensitivity to sound, velocity, and head-body angle. Indeed, responses in the presence and absence of auditory stimulation showed no significant difference (paired *t*-test, n.s. for each unit separately, see Additional file 14). To check the independence of location sensitivity from velocity (or head-body angle), we computed the matrix of mean firing rates as a joint function of spatial position and velocity (or head-body angle). We then computed a rank 1 approximation of this matrix using nonnegative matrix factorization (see Additional file 15). A good rank 1 approximation to the observed firing rates implies that the two parameters cause largely independent changes in the firing rate of the unit. We then verified that this rank 1 approximation explained an appreciable amount of the structure of the data (see “Methods” for details). In general, the rank 1 approximation to the observed firing rate accounted for a similar amount of variability as expected from surrogate data, verifying that for the units presented here, location sensitivity was indeed independent of velocity and of head-body angle.

The second type of units illustrated here (Fig. 6i–l) showed sensitivity to the angle between the head and the body of the rat, extracted from the video stream by the data analysis pipeline (see “Methods”). Such units were found both in AC and in Ins. We tested the significance of the dependence of the firing rates on the head-to-body angle units. The units in Fig. 6i and j responded strongly when the rat was looking to the sides compared to the front (1-way ANOVA, F(11,54860)=221, *p*=0, and F(11,35050)=63.3, *p*=8.27×10^−141^ respectively). The units in Fig. 6k and l responded strongly only when the rat was looking towards one side (1-way ANOVA, F(11,26372) = 33.3, *p* = 3.23×10^−71^, and F(11,34830) = 31.7, *p* = 7.99×10^−68^ respectively). To control for confounds, we checked that their sensitivity to the relative head direction was not a consequence of their sound responses (paired *t*-test between angle-dependent mean firing rate in the presence and absence of sound, n.s. for each unit separately, Additional file 16). The head-body angle sensitivity was independent of location in the RIFF, and of the absolute angles of the rat body and head in space (see Additional file 17 for full results). The unit presented in Fig. 6h is the same as that presented in Fig. 6l. It showed sensitivity to both rat location and head-body angle, but those sensitivities were independent of each other.

## Discussion

Jointly studying brains and behavior requires compromises. Virtual reality setups have been developed to flexibly study neural activity during behavior [14–23]. Such systems allow for the stable acquisition of high-quality data in head-fixed animals [20] and can integrate imaging techniques that are harder to apply in freely moving animals [24]. Still, virtual reality is an approximation, may provoke unnatural movement patterns [25, 26], and evoke neural responses that differ from those observed in real environments [18, 27].

Many high-throughput systems for studying freely moving animals have been developed to explore learning and plasticity [28], navigation [19, 29–32], exploration [33], social interactions [33–42], and other behaviors [34, 35, 43]. Some can even track multiple individuals simultaneously.

Only a few behavioral environments include wireless recording of neural activity from freely moving animals [44–47]. The novelty of the RIFF in this emerging field lies in its integrated approach allowing a rich repertoire of behavioral tasks combined with wireless electrophysiology. The arena is relatively simple due to its circular symmetric shape, while still allowing a large range of sophisticated experiments that provide extensive behavioral freedom for the animals. The relative simplicity of the environment makes the interpretation and modeling of animal behavior simpler than the interpretation of behavior in ethological environments, providing an intermediate level of experimental complexity between restricted and fully unconstrained behaviors.

The RIFF is a fully automatic, high-throughput, high-resolution environment for collecting detailed online behavioral information together with tightly synchronized neuronal recordings. The RIFF is designed to support decision-making tasks that involve a large set of possible actions and that take advantage of the relatively large arena and its ability to run long-lasting automatic tasks, reducing manual interventions.

The design of the RIFF was kept as modular as possible so that different choices for hardware and software can be easily accommodated. We concentrate here on the design principles and the logic implemented by the RIFF, including the integration of behavior and electrophysiology into a complete environment for studying brain processes in freely moving animals, while providing substantial amount of information on the current implementation in the “Methods” and supplemental information. As an example, BPods (Sanworks, Rochester NY, USA) implement state machines that interface with external hardware. They can replace the Campden-Lafayette control equipment and software that we used to control the ports, interfacing directly with a Matlab main program. Similarly, Bonsai (https://bonsai-rx.org/) is a visual programming language for controlling real-time setups. It could be used to replace some of the proprietary control software we used, and potentially replace Matlab in the main loop of the RIFF.

Tasks for which the RIFF is less suitable are for example tasks that involve simple behaviors for which the RIFF is too complex. These include classical paradigms such as fear conditioning which is often performed in small enclosures [48, 49]. Similarly, behaviors that require substantial amount of manual interventions would be less appropriate for the RIFF. Such are for example novel object recognition tasks, which is often performed through short trials [50].

The current implementation has a limited range of peripheral equipment, limiting again some applications. For example, we evaluate here a neural-network-based pose-estimation algorithm optimized for our hardware. Substantial progress has been made recently in the tracking of animals and body poses in real time [51–53]. Published algorithms require additional training to the specific setup at hand so that it is unclear whether they have any advantage for the RIFF in its current implementation, using a single camera. However, while high-level behavior identification [54–56] can be achieved in the RIFF, it would require an upgraded version of the video processing component (using multiple and/or faster cameras). In the same vein, the use of the RIFF is limited in studies of fine motor performance, and it is currently not designed for somatosensory, olfactory, or visual tasks.

However, the modular design of the RIFF allows additional elements to be easily added—for example, while it can provide simple visual cues, in addition to auditory cues, for guiding behavior already in its current implementation, more complex visual tasks may require the addition of video screens in appropriate locations. Other sensors can be added as well, such as motion capture setups [57, 58] and a more powerful video processing component. The RIFF can be also easily reconfigured by physically moving ports around, changing the number and position of interactive ports. For example, it can be modified to the geometry required in five choice serial reaction time task, 5CSRTT, [59–61].

Finally, the RIFF can be used with high-density electrodes that have hundreds of contacts [62] coupled with commercially available wireless loggers (e.g., SpikeGadgets Neuropixels headstage).

We demonstrated here the tracking of behavior and electrophysiology in a precise manner in time and space. Animals learned successful behavioral strategies very quickly. This is surprising, as learning complex tasks sometimes takes weeks or months [63–65]. Potentially, the large environment and the multiple possible actions available at each moment encouraged more extensive exploration [66, 67], leading to faster learning.

To the best of our knowledge, we provide here for the first time evidence for neurons in Ins whose responses depended on the location of the animal in space. Ins receives direct projections from the primary division of the auditory thalamus, the ventral medial geniculate body [68], and has neurons with frequency-tuned, short-latency responses [13], but in rats it is spatially separate from the primary auditory cortex [12]. The location-sensitive neurons described here are therefore intermixed with neurons with response properties similar to neurons in the primary auditory cortex. The evidence provided here adds to other recent studies that described location-sensitive neurons in many structures outside the hippocampal system [69, 70], including visual [71], somatosensory [72], retrosplenial [73], and premotor [74] cortices.

Several frameworks for the design of behavioral tasks in neuroscience exist [75–77]. In contrast to these systems, the RIFF is a complete, well-defined behavioral arena, not just a control framework. Moreover, the RIFF can be customized to uniformly address questions in behavioral neuroscience in different animal models. Elements of the arena directly interfacing with experimental animals can be down-sized to fit smaller animal models (in practice, by replacing the interactive ports and downsizing the rewards). At the same time, the logic of the RIFF as illustrated in Fig. 3a need not be modified at all, keeping the high temporal resolution and multiple data streams intact. Such an arena would fit mice and gerbils, a common animal model in comparative auditory studies [78–80].

## Conclusions

The RIFF was designed for maximal flexibility in the investigated behaviors while keeping a tight synchronization between behavior and electrophysiology, integrating information from many sensors to react to animal actions at latencies that are limited only by the efficiency of the “tight loop” of the control program. A number of results presented here show the potential and power of this approach. Rats learned rather complex behavioral contingencies very fast (1–2 days of training), and we identified unexpected connections between behavior and neural activity. We present examples of neurons in primary and high-order auditory areas that show sensitivity to non-auditory features of the tasks. The highly detailed data collected from each animal allowed us to control these findings for a large number of confounds, such as simultaneously presented sounds, head and body directions, and animal velocity.

## Methods

### Behavioral setup

The arena, 160 cm in diameter, is composed of 18 modular sections (28 cm width, 50 cm height—aluminum skeleton and 5 mm thick black matte Perspex sheets; one section is illustrated in Fig. 2b and Supp. Additional file 2 illustrates many of the details described in this section). The floor is made of two sheets, tightly connected by screws. The arena is mounted on a 5-cm high supporting structure, of the same size and shape of the floor sheets with depressions for the connection between the two sheets as well as for the insertion of gridded floors (for possible foot shock punishment). The arena is electrically grounded with a cable running around the outer perimeter near its bottom.

Each of the 18 sections contains three panels where additional equipment can be inserted. In the RIFF, the outer panels of every third section contain two nose-poke ports (Fig 2; a total of 12 ports in 6 sections, SPECIAL.090-SE v1.0, LaFayette-Campden Instruments, Loughborough, UK). One of the two ports in each section is connected to a liquid pump (Model 80204, LaFayette-Campden Instruments) and the other to a pellet dispenser (Model 80209, LaFayette-Campden Instruments). In addition, all 12 ports are connected to an airpuff valve (Series 3 miniature inert liquid valve, Parker). At the top of each panel with a port, a free field speaker (MF1, Tucker Davis Technologies, Alachua, FL, USA) is mounted in a custom-made holder with an adjustable angle. Such a section, with its two ports and two loudspeakers, is termed an “Interaction area” (IA) in the paper.

### Auditory stimulation

In both tasks described here (St+ task and L/D task, described below), a small set of sounds was used. These sounds were synthesized ahead of time and stored in computer files. Pure tone stimuli were synthesized in Matlab. Word-like stimuli were created using the following procedure. Words were selected from free online recordings (http://shtooka.net). They were then processed using the STRAIGHT Vocoder [81] and Matlab. For sounds in the St+ task, the spectrotemporal envelope was extracted by STRAIGHT and shifted above 1 kHz. For the sounds in the L/D task, the spectrotemporal envelope extracted by STRAIGHT [81] was shifted and stretched along the frequency scale, so that the envelope levels at frequencies 0.1 and 20 kHz of the original sound were shifted to frequencies 1 and 40 kHz (with linear interpolation on a logarithmic scale in between). The resulting spectrotemporal envelope of each sound was used to resynthesize it using the original pitch contour.

The acoustics of the arena were studied in detail [82]. In short, the arena produces two major reflections, one from the floor and the other from the wall behind the animal. The dominant reflection from the floor creates spectral modulation with a period of 5 kHz and depth of about 5 dB. Absolute sound levels at 0 dB attenuation were about 100 dB SPL at 2 kHz, going down to about 85 dB SPL at 20 kHz. Sound levels were rather stable as a function of location in the arena (varying by <5 dB between the center of the arena to the walls) and of loudspeaker (standard deviations across loudspeakers at the center <5 dB; at the wall <8 dB). No correction was applied for this variation.

### The video imaging system

A custom video system tracked the trajectory of the animal in real time at 30 frames/s (Additional file 8). The localization jitter was less than 30 ms and a compact storage format resulted in data volume of 4.3 GB/h. The system robustly tracked rats of different sizes and was unaffected by the presence of a head implant.

#### Hardware

A monochrome camera (DMK 33G445 GigE, TheImagingSource, Bremen, Germany) was mounted on the ceiling above the center of the arena. A wide-field lens (T3Z3510CS, Computar, North Carolina, US) covered the whole arena. A LED ring (50 cm in diameter, CN_R64, NanGuang, Chenghai, China) placed around the camera provided a uniform diffuse illumination (Additional file 18a). The luminance of the ring was set to the minimal level that was sufficient for exposure time of 25 ms.

The camera was connected to a computer running the Windows operating system with a GigE cable that supplied power and transmitted the grayscale 1280×960 pixel images. The triggering pulses were sent from the camera by a Hirose cable to the digital input section of a multifunction device (RX8, Tucker-Davis Technologies). The RX8 subsampled the incoming trigger train from 30 to a 1 Hz pulse train and output it to the main recording unit (AlphaLab SnR, AlphaOmega). These 1 Hz triggers also powered a LED that was placed outside the arena but inside the field of view of the camera (Additional file 18b). The LED state was used to synchronize the images with the rest of the recorded data during the experiment.

#### Real-time image acquisition and rat localization

A custom graphical user interface (programmed in Matlab) was used to launch the tracking program and monitor its activity in real time (Additional file 18c; https://github.com/jniediek/RIFF_publication/tree/main/acquisition/camera_tracker). A single background image of the empty arena was acquired during the initialization of the tracking program. The real-time tracking routine executed an acquisition-localization tight loop: Every time a new image was acquired from the camera, the rat was located by subtracting an image of the empty arena from the current image of the arena with the rat inside. The difference image highlighted the area where the rat was located. The location of the rat was estimated by fitting an ellipse to the rat pixels and computing its center of mass. The *x* and *y* coordinates of the estimated rat center of mass were sent to the computer that controlled the experiment over an Ethernet connection and were also stored as metadata.

To reduce data storage, the video stream was saved in a compact form. Each rat image was cropped around the rat center of mass, since only this area was expected to change from one frame to the next. The original image could be approximately reconstructed by placing the cropped rat image on top of the background image at the location indicated by the coordinates of the center of the rat, stored in the metadata file. In addition, one of the corners of the bounding box was replaced by a small rectangle that showed the LED (Additional file 18d). The images and the metadata were stored in a compact database at the rate of 4.3 GB/h.

### Integration and the main control program of the RIFF

The current implementation of the RIFF includes four computers with separate roles, with the goal of providing a high modularity to the system. The computers and their associated peripheral components are schematically illustrated in Additional file 1, each computer and its associated peripherals drawn in the same color. The four computers are the behavioral response computer (Additional file 1, item 8); the video processing computer (Additional file 1, item 14); the timing and electrophysiology computer (Additional file 1, item 17); and the master computer, running the main control program (Additional file 1, item 9).

The behavioral response computer, interfaces with the hardware (Additional file 1, items 2-7) that controls the ports through a combination of commercial software (ABET II, LaFayette-Campden Instruments, Loughborough, UK) and a custom-written Matlab program (The MathWorks, Inc., Natick, MA, USA) on the master computer. A data file that includes all the events on all ports is produced and saved on this computer.

The video processing computer (Additional file 1, item 14) was described above.

The timing and electrophysiology computer interfaces with the electrophysiological recording systems (both the wireless and the neurologger versions, Additional file 1 items 15-18). In addition, the AlphaLab SnR (Additional file 1 item 15; Alpha Omega, Nazareth, Israel) that is used by the TBSI system for data collection has a set of digital inputs that are used to set a common time base for all equipment, as will be described below. We used the same digital inputs even when the electrophysiological data was collected on the neurologger.

The master computer controls the audio presentations. During the experiment, all sounds were loaded to memory. When a sound had to be presented, it was transduced to voltage signals by a sound card (M-16 and MADIface USB, RME Audio Interfaces, Munich, Germany), attenuated (PA5, Tucker Davis Technologies, Alachua, FL, USA), and then played through stereo power amplifiers (SA1, TDT) (Additional file 1, items 19-21) connected to loudspeakers (TDT MF1, Additional file 1, item 1). Two dedicated outputs of the sound card were used for timing pulses at the precise time of sound onsets.

The master computer interfaced with the behavioral system through a digital I/O card and a connector block (NI PCI-DIO-96 and SCB-100, Additional file 1, item 10) that reads some of the information coming from the ports and outputs digital signals to other lines that cause the behavioral computer to provide reward. The master computer is also controlling a muti I/O Processor (TDT RX8, Additional file 1, item 12) which is used to produce and shape timing signals for synchronizing the whole system.

The digital lines on the AlphaLab SnR are used to collect some of the timing pulses that are generated by the rest of the system, producing a common time base on which all the events in the system can be synchronized. For synchronization with the behavioral control computer, the Multi-I/O Processor produces a periodic timing pulse which is recorded on the behavioral response computer as well as on the AlphaLab SnR. For synchronization with the video camera, the strobe signals from the camera that indicate frame captures were channeled to the Multi-I/O Processor, downsampled by a factor of 30, and then channeled to the AlphaLab SnR and also to a LED captured by the camera. These LED signals were used to synchronize the video stream with the rest of the system. Finally, an audio channel carrying timing pulses was also channeled to the AlphaLab SnR, allowing the fine synchronization of sound onsets with the electrophysiological data. These digital lines are sampled at 40 kHz, and therefore all events can be synchronized to a precision better than 25 µs.

The heart of the RIFF is the main loop that implements the task logic by interacting with the surrounding hardware (video camera, ports, and the sound card). By modifying the contingencies controlled by this loop, a large variety of tasks can be implemented in the RIFF. The main loop for both tasks that are described below is provided (https://github.com/jniediek/RIFF_publication). Instructions on how to modify the main loop of the L/D task (described below in detail) in order to implement other contingencies is provided in the wiki section of the github repository (https://github.com/jniediek/RIFF_publication/wiki/Your-mission).

We tested the timing performance of the main loop. The running time of the main loop of the RIFF, when it did not have to wait for information from the camera, was <8 ms (median 1.6 ms, but the distribution was double-peaked with a second peak at about 4.5 ms; range 1–8ms, *N*=71,388; Additional file 19a). In consequence, the RIFF software in its current implementation can process video frames at rates of up to about 120 Hz.

When interacting with surrounding software, some additional latencies incurred. For the real-time control of the RIFF, the most important latency was between image acquisition and the moment it became accessible to the main control program for further processing. All latency measurements were performed by a BPod system (Sanwork LLC) which recorded hard triggers from the camera and other hardware components, as well as soft triggers at selected code locations.

All data is illustrated visually in Additional file 19. The delay from the moment a frame was available on the camera (not including exposure time and sensor readout time, which are determined by the specific hardware) and the time it was read by the Matlab program on the video processing computer was 6.2 ms with a very low jitter (std=1.1 ms, range 4.6–10 ms, *N*=164; Additional file 19b). The Matlab logic that extracted the *x* and *y* coordinates of the center of the rat added about 4 ms without increasing the jitter (median latency 10.3 ms, std=1.1 ms, range 8.4–16.1 ms, *N*=164; Additional file 19c). The transfer of these coordinates to the main control program lengthened the latency to 39.5 ± 11.8 ms (as reported above, range 11.3–61.5 ms, *N*=186; Additional file 19d) Thus, the Ethernet connection between the video processing computer and the main control computer added almost 30 ms to the latency but also increased the jitter.

Once the information reached the control computer, the main control loop could react to it. For example, within the main loop, another 15 ms was needed to characterize the location of the rat and, when necessary, select a sound to play. This additional latency had a low jitter, since the total jitter did not change appreciably at this stage (total latency from frame acquisition: median=55 ms, std=12.8 ms, range 12.8–78 ms, *n*=94; Additional file 19e). Finally, the latency to sound presentation was about 180 ms, largely because of the time required to update the attenuator but, with only a small increase in the jitter (total latency from frame acquisition: median 237 ms, std=16.9 ms, range 204–287 ms, *n*=94; Additional file 19f).

### Post-experiment data processing

After the conclusion of each experimental session, data were processed by a custom, automated pipeline written in Matlab (tested with version 2020b). The full software of the analysis pipeline is available (https://github.com/jniediek/RIFF_publication/) and instructions on how to run it on real data examples collected in the RIFF are provided in the wiki (https://github.com/jniediek/RIFF_publication/wiki/The-analysis-pipeline) [11].

Data processing started with the extraction, alignment, and merging of the time stamps from all data acquisition devices. Time stamps were generated for changes in internal states, the acquisition of video images, behavioral events, sound presentations, and neural recordings. The digital lines of an AlphaLab SnR system (Alpha Omega) were used as the main synchronization hub for aligning the triggers coming from all devices. Each device that generated timing triggers had at least one of those triggers also channeled in parallel to a digital line on the main synchronization hub, and the timing events that were co-registered on both devices were used to align all other events from that device on a common timeline. Thus, the output files of the SnR system were used as the main timing files during post experimental data processing. All behavioral events (nose pokes, food/water delivery, airpuffs) as well as the video images were parsed, and the extracted information stored in Matlab files. Finally, the neural recordings were processed, detecting and sorting spikes (see next section).

#### Spike sorting

We developed a pipeline that processes an experimental session of 3 h in about 10 min, based on the Kilosort2 spike sorting program (https://github.com/MouseLand/Kilosort2). The processing was predominantly autonomous, requiring only a few minutes of manual curation for every session.

Spike sorting began by removing common modes from the neural recordings—the mean activity of all spatially proximal recording channels, such as all channels on a single shank of a silicon probe (Additional file 7a.1-2). Large artifacts were identified by computing the standard deviation over electrodes at each point in time, and locating peaks in the resulting time series. Such artifacts were mostly generated by rat movements. Signal sections around each of these peaks were zeroed by multiplication with a 10-ms window with 2-ms rising and falling edges (Additional file 7a.3). The total recording duration that was zeroed during a typical experimental session was about 5%.

Spikes were detected and clustered by Kilosort2. In order to process large amounts of data, we developed a fast and almost automatic pipeline, which can be complemented by detailed manual curation where necessary. Kilosort2 was used to produce the initial set of candidate spike clusters. Automatic rejection of noise clusters was performed according to two criteria (Additional file 7b): The dominant channel of the cluster template has exactly one minimum and one maximum in its voltage trace, and on each electrode the voltage at the template endpoints had to be close to 0 (in consequence, confining expected spike duration to <1.8 ms). For each unit that passed this test, an inter-spike-interval histogram was computed alongside the amplitude and firing rate histograms, heat-map of raw spike waveforms, and smoothed time series of spike amplitude and firing rate (Additional file 7c). All those were printed to an image file that summarizes the quality of that unit.

Lastly, a manual labeling interface allowed the experimenter to sort the units into well isolated ones, multi-unit clusters or noise (https://github.com/jniediek/RIFF_publication/wiki/Manual-tagging). This interface displayed the images with the quality evaluation information and stored the user-inserted labels with the data. A typical recording session of 1–2 h with 32 electrodes produced typically a few tens of non-noise units, so that the manual labeling could be completed in several minutes.

#### Feature extraction from rat images

Pose information was extracted from the saved video stream, offline, in two steps: First, the nose, the base of the neck and the base of the tail were located on the image by a convolutional neural network (Additional files 4a and 5). Then, the relevant angles were computed by geometric calculations (Additional file 6, green arrow represents the body direction). The neural network was trained de novo, including data labeling, model training, and time optimizations of the inference process.

We used the CNN, without retraining, on eight rats (4 rats in each in the St+and in the L/D tasks). Instructions on how to use and retrain the CNN are provided at https://github.com/jniediek/RIFF_publication/tree/main/misc/train_direction_tagger.

##### Network architecture

The input to the model is a square grayscale image of the rat, as produced by the rat tracking module. The output consists of three pairs of Cartesian coordinates, representing the three points of nose, neck, and the base of the tail on the rat image. The model is thus a multivariate regressor that predicts 6 continuous variables.

We used a custom convolutional neural network architecture, following the recent advances in data-driven computer vision (Additional file 4b). Six convolutional layers are used, each followed by a batch-normalization layer and a rectifier non-linear activation function. A max-pool layer operates after every second convolutional layer. The last layer is a linear readout from the previous activation map stack. The network has 0.3 million trainable parameters.

##### Database creation

We used a custom programmed graphical user interface (written in Matlab) to manually mark the locations of the nose, neck, and base of the tail on each image of the training set. The final training database included 1500 labeled images of 4 rats of different ages, with and without a head implant.

##### Network training and inference enhancements

The training database was augmented to produce additional training examples and to emphasize the irrelevant degrees of freedom in the image space. The augmentation included rotation of images by multiples of 90°, horizontal mirror flips, adding small amounts of noise to pixels or labels, and rigid translations of images by a few pixels along the horizontal and vertical axes. The labels were re-adjusted for each augmentation type to correctly represent the updated body part location. This augmentation procedure increased the original dataset by a factor of 64, from 1500 to 96,000 training examples.

The network was initialized with the default Kaiming distribution [83], trained with the Adam optimizer [84] on 256 images in each batch. The input images were subsampled to 50×50 pixels. The network was trained for 2 h on a NVIDIA GTX 1080Ti GPU (NVIDIA, California, USA).

After the training was completed, the network could be used to predict the labels of previously unseen images. Prediction used image augmentations that resemble those used during the training: An input image was processed 4 times, rotated by 0°, 90°, 180°, or 270°. The predicted 4 labels were corrected for the corresponding image rotation and averaged to obtain the final label for the original image. The high temporal correlations of the image stream were leveraged to increase the accuracy by smoothing the labels with a uniform window of 7 frames, removing large, biologically implausible jumps.

An hour of a typical experiment produced ~10^5^ images, which were labeled by the trained model in ~20 s.

##### Computation of allo- and ego- centric directions

Once the locations of the nose, neck, and the base of the tail are predicted by the network, they are used to calculate the direction of various body parts. The main body direction is defined as the vector from the base of the tail to the base of the neck. The head direction is defined as the vector from neck to the nose point. The head turn angle is calculated as the difference between the head direction and the body direction, with 0 corresponding to the three points being along a single line. The range of head turn angles was typically between −30° and 30°.

##### Network performance

We evaluated the accuracy of the network on a set of manually tagged images that were excluded from the training set. The accuracy was defined by the mean distance of the predicted points from the manually tagged points on the rat body (Additional files 20 and 21). The mean localization errors of the head, neck, and tail were 0.62, 0.66, and 0.62 cm, respectively (see Additional file 22).

### Final database formats

The data processing pipeline parsed each experiment into a set of data files, metadata descriptors, and statistical visualizations. This format is uniform and allows an abstraction of the experiment parameters such as the type of neural recording system, the stimulus types, and so on. All behavioral and neural events are stored in a lossless manner, allowing maximal temporal resolution. The storage footprint of this format is about 12 GB/h. The format is described in the repository (see information in https://github.com/jniediek/RIFF_publication/wiki) [11].

For rapid analysis and visualization, a temporally coarse-grained version is generated by discretizing the timeline into a 10-Hz grid, and aggregating the separate data files into a single data table (stored in one file). This format uses about 1% of the storage of the raw storage (<0.1 GB/h). Each data row in the resulting data table has a time stamp that indicates its start time on the time axis of the original data. These time stamps can be used as indexing variables for tracing the data back to the high-resolution files.

### The experiment visualizer

All processed data from an experiment can be loaded into an interactive user interface that presents the recorded experiment as an interactive movie (at a rate of 10 Hz, see Fig. 3f). The raw neural data is loaded along with the detailed time information of the sorted spikes, making it possible to view both the firing rate of each unit and the times of individual spikes with a 1-ms resolution. The full software of the visualizer is provided in the software repository (see https://github.com/jniediek/RIFF_publication/wiki for instructions) [11].

The visualizer was used for multiple purposes. During the development of the experiment, replay of the experiment with 100 ms resolution made it possible to verify that the experimental logic was correctly implemented and executed by the hardware, and the rat indeed performed the required sequence of actions. The visualizer was also used for verifying the collected data; the temporal resolution of the visualizer makes it possible to detect potential data collection malfunctions such as synchronization errors. During data analysis, the visualizer was used as a tool for understanding fine details of rat behavior and for observing the progress of the learning process. Lastly, the visualizer can plot the raw neural traces at any time point of the experiment, making it possible to observe the relationships between spiking activity and other events in the RIFF. Figure 3c and f show images produced by the visualizer. The visualizer was used to generate Additional file 21 and 23.

### Animals

The experiments were carried out in accordance with the regulations of the ethics committee of The Hebrew University of Jerusalem. The Hebrew University of Jerusalem is an Association for Assessment and Accreditation of Laboratory Animal Care (AAALAC) accredited institution.

Seven adult female Sabra and Sprague Dawley rats (*N* = 5 and *N* = 2, respectively, weight: at least 200 g) were used for the experiments described here (Envigo LTD, Israel). All efforts were taken to create a low-stress, rat-friendly living environment enabling experimental animals to freely express their innate behaviors. Upon arrival, animals were housed in groups (2–3) or individually (depending on experiment) in the same SPF room in which the RIFF is situated and where all experiments took place. The temperature (22 ± 1 °C) and humidity (50 ± 20%) were controlled and room was maintained on a 12-h light/dark cycle (lights on from 07:00 to 19:00 h).

### Behavioral training

For the first 7–10 days rats were habituated to the experimenter. During this period, rats were handled frequently and introduced to new types of foods and flavored water which were later used as rewards in the experimental arena. The procedure was aimed to decrease stress and prevent neophobia towards rewards given during behavioral training. Rewards consisted of palatable 45 mg food tablets in six flavors: basic company flavor, banana, bacon, chocolate, peanut butter, and fruit punch (diet AIN-76A: RodTab45MG; RodTabBan45MG; RodTabBcn45MG; RodTabChoc45MG; RodTabPbtr45MG; RodTabFrtP45MG5TUL, TestDiet, Richmond, IN, USA). Rats received also 4 types of fluids: mineral water, 4% sucrose in mineral water, 0.1% saccharine in mineral water, and 1:1 mixture of 4% sucrose, and 0.1% saccharine solutions (sucrose and saccharin, Sigma-Aldrich, St. Louis, MO, USA).

In order to motivate animals to work for food, rats were food-restricted up to 85% of their ad libitum body weight. Rats were subjected also to mild water restriction before behavioral sessions (4–12h). Experiments were carried out 5 days a week. During the weekend, animals had free access to standard rodent food and unsweetened mineral water.

#### Multiple-strategy task (St+)

In this task, rats had to move to specific locations at defined times indicated by distinct sound sequences, in order to obtain rewards. The experiment consisted of a repeated iteration over three events: Attention (ATT) - Target (TGT) - Feedback (FDB). Each event was associated with a specific sound that was played whenever the event occurred. The paradigm is described in detail in Additional file 3a. The ATT event consisted of the presentation of an ATT sound—a 50-ms broadband 2 kHz periodic sound that was repeated 5 times at intervals of 0.3 s. It was played from all 12 speakers simultaneously. The position of the rat was determined 2.5 s after the ATT event, and one of the IAs was selected as the target location. The selection was done according to the current and previous position of the rat (see below for details). Then a TGT sound was presented from the target location. Each IA was associated with a specific TGT sound. The TGT sounds consisted of a sequence of six 50-ms-long narrowband sounds with inter-sound intervals of 250 ms covering a range of 1–16 kHz. Six different permutations of those sequences were assigned as target sounds to each IA for the whole experiment (target area 1: 1, 1.7, 3, 5.2, 9.2, 16 kHz; target area 2: 1.7, 16, 1, 9.2, 5.2, 3 kHz; target area 3: 3, 1.7, 1, 16, 9.2, 5.2 kHz; target area 4: 5.2, 3, 1.7, 1, 16, 9.2 kHz; target area 5: 9.2, 16, 5.2, 1, 3, 1.7 kHz; target area 6: 16, 9.2, 5.2, 3, 1.7, 1 kHz). After the TGT event, the rat had to nose-poke into one of the two ports at the target IA. Nose-poking within 20 s after the TGT event was rewarded by food pellets or water, depending on which of the two ports of the IA were poked. Reward was provided only if the first nose poke was in the correct IA, and only to that first nose poke. The first nose poke (in a correct or wrong location) elicited the FDB event at the target IA, consisting of a sound indicating whether the location was correct or not (word-like stimuli derived from the word “correct” or “mistake”, respectively). A timeout (20 s without poking after a TGT event) was also followed by an error FDB sound. After each FDB event, an inter-trial period of 3 s led to the next ATT event.

The target locations were selected in the following way. The arena was subdivided (Additional file 3a) into A zones, in the immediate vicinity of the IAs; B zones, adjacent to the A zones but 17 cm towards the center; C zones, separating the A/B zones of nearby IAs; and a large D zone in the center of the arena. The A and B zones were slightly wider (33 degrees) than C zones (27 degrees). When the rat was in the D zone, nothing happened. If the rat was in one of the A/B/C zones, an ATT sound was presented (3 s following the last FDB event). The rat had to make a choice where to go within the next 2.5 s. “A” choices consisted of the rat remaining in the same A zone in which it received the last reward, in which case the target was presented from the next A zone in the clockwise direction, or by a move to another A zone, which was then selected as the target. In these cases, the rat got 1 reward (pellet or liquid). “B” choices consisted of a move to a B zone, in which case the next target consisted of the adjacent IA and the reward was 3 times larger than when making “A” choices. The rat could therefore cycle between a B zone and the adjacent IA ports, harvesting rewards from the same port. Finally, the rat could move to a C zone, in which case a random target was selected and the reward was randomly chosen at 1–4 times the reward of the “A” choices. In the data shown here, rats learned an “A” strategy—moving around the arena from one IA to its neighbor, circling the arena. In fact, the rats rapidly learned the timing structure of the task, and often moved to the next A zone before the ATT sound was presented (Fig. 4d).

The rats were trained in three phases. During phase 1, rats were trained to poke for reward. Multiple food and water restricted rats were placed together in the arena for about 12 h for five consecutive days. The sounds were not presented at this point. The rewards were delivered in a fixed ratio (FR) schedule. The number of pokes needed to get reward was increased every day as follows: FR2, FR7, FR10, FR12, and FR15.

In phase 2, rats established instrumental and Pavlovian associations with relevant sounds and their sequences. Rats were trained individually, each during a 70-min-long session performed once a day. The training was carried out for 30 days. The sound level was increased gradually to avoid suppression of behavioral responses and to introduce animals gradually to the relatively high SPL of the sounds during the main experiment. Each day, only 1 IA was accessible for a rat during the session. Interactive ports in all other 5 areas were closed by custom blinds. Consecutive sessions were carried out in areas 1 to 6 and such a cycle was repeated 5 times. There were 3 types of sounds presented in this phase. The rat started the trial by poking into the accessible port. Each poke (photo beam break) evoked the 50-ms broadband 2 kHz periodic sound. After five successful pokes with sound, the sixth poke triggered reward delivery (1 unit) and immediately the target sound (TGT) was played from the two speakers above the accessible IA. The TGT sound was followed by 1 s of silence and the word-like stimulus derived from the word “correct” played from two speakers above the accessible IA. An obligatory 2-s interval followed before the RIFF reacted again to nose pokes of the rat. Thus, during that stage, while sounds were associated with reward, it was the nose poke that triggered the sounds rather than the reverse (as in the main task).

Phase 3 was the main experiment as described above. Rats performed a 70-min-long experimental session once every day.

The behavioral data in Fig. 4 was collected during the first 2 days of Phase 3 of the task. However, the electrophysiological data shown in Fig. 5 was collected after one additional refinement of the task (phase 4, Additional file 9). In randomly selected 10% of the trials, a warning sound was presented (the phrase, composed of word-like stimuli: “do not go,” played from all speakers). The rats had to avoid accessing the ports in order to avoid airpuff, starting 1.5 s after the warning sound (to let the rat retract its nose from the port within in case it was poking when the warning sound was presented). An airpuff was triggered by a nose poke for pokes that occurred during the next 3.5 s. If the rat successfully avoided poking any of the ports, a safety sound (the phrase, composed of word-like stimuli: “it’s safe”) was presented from all speakers. Otherwise, an airpuff was delivered and the phrase “you failed” (composed of word-like stimuli) was presented from all speakers. After safety or punishment sounds, a new trial was initiated with ATT sound.

#### Localization/discrimination task (L/D)

In this task, each of the IAs was associated with a sound. The sounds were 200-ms excerpts of 6 different words (the word “here” in 6 languages: English, French, German, Italian, Polish, and Russian) in a female voice, processed as in the “ Auditory stimulation” section. The rats had to go to a central circular area to initiate a new trial (Additional files 3b and 10). When a crossing into the central area was detected, one of the 6 IAs was randomly selected by the program and its associated sound was played once every 2 s from both speakers for up to 10 times (20 s in total), or until the rat poked in any port. A poke in one of the two ports of the IA from which the sound was played resulted in a reward (food or water, according to the poked port). A wrong poke or a timeout (20 s without a poke) resulted in the termination of the trial.

The rats were habituated to the RIFF during 2 nights (24 h in total). During habituation, the RIFF was divided into 6 equal sectors centered on the IAs (Additional file 3b). Rats were placed in the RIFF and were able to explore freely. Whenever the rat entered an active sector, the associated sound was played from the speakers of the IA in the sector. The sound repeated every 2 s until the rat poked in one of the ports of the IA and got a reward, with a timeout after 2 min. After a nose poke, the current sector and its two neighbors became inactive and the three other sectors became active, so that the rat had to cross to one of the three sectors on the opposite side of the RIFF in order to initiate a new interaction.

Following the habituation, the rats were exposed to increasingly stricter versions of the main task in 12-h overnight daily sessions. For the first 6 training sessions, the rats were allowed to poke in more than one port before trial termination (10 pokes in the first and second sessions, decreased to 6 and 4 for one session each, 2 pokes for 2 sessions, then down to 1 for the rest of the experiment). In addition, the requirements for initiating a trial became stricter: on the first training session, the central area had a radius of 50 cm, on the 2nd session of 40 cm, and from the 3rd session on the central area had a radius of 30 cm. Before electrode implantation rats were switched from 12-h overnight sessions to 3-h morning sessions.

### Electrodes

Rats were chronically implanted with 32-channel silicon probes (ASSY-116_E-2, Cambridge Neurotech, UK). Prior to implantation, thin flexible ground wire was soldered to the electrode’s PCB ground contacts. The electrodes were aligned and glued to the microdrive shuttle with epoxy (Nano Drive, Cambridge Neurotech, UK). The microdrive with the electrodes and connector were held by a custom holder attached later to a stereotaxic apparatus for implantation. The moveable parts of the microdrive were covered with paraffin oil to prevent possible leak of dental cement between them. Before implantation, the electrodes were cleaned by washing them with a 4% tergazyme solution in purified DDW water (Alconox, Jersey City, NJ, USA) and afterwards carefully washed in purified DDW to remove all residues of tergazyme. Before implantation, the electrodes and the holder were disinfected in a UV sterilizer.

### Surgery

The implantation of the silicon probes was performed in two stages: (1) preparation of the base for the implant and (2) implantation of microelectrodes into the brain tissue.

#### Preparation of the base

Rats were initially anesthetized in an induction chamber with sevoflurane (8% in oxygen, Piramal Critical Care Inc., Bethlehem, PA, USA). The head was shaved and they were placed in a stereotaxic instrument with a mask for gas anesthesia (David Kopf Instruments, CA, USA). Sevoflurane concentration was slowly adjusted to a level of 2–2.5% and maintained at this level throughout the surgery. A surgical level of anesthesia was verified by the lack of a pedal-withdrawal reflex and slow, regular breathing rate. Body temperature was controlled by a closed loop heating system with a rectal probe (Homeothermic Monitoring System, Harvard Apparatus, MA, USA). The eyes were protected with sterile eye drops for dry eyes (Viscotears Liquid Gel, Carbomer: polyacrylic acid 2 mg/g, Berlin, Germany), and the skin on the head was disinfected with a povidone-iodine solution (10%, equivalent to 1% iodine, Rekah Pharm. Ind. Ltd., Holon, Israel). To prevent postoperative pain, rats received during the surgery subcutaneous injection of Carprofen 50 mg/ml (5% W/V) in a dose of about 12 mg/kg (Norocarp, Norbrook Laboratories Limited, Newry, Co. Down, Northern Ireland).

A 1.5–2-cm longitudinal cut of the skin on the head was made and the dorsal surface of the skull was exposed. The opened skin was stretched and the eyes were closed. The left temporal muscle was pulled away to expose also the lateral surface of parietal and temporal bones. The connective tissue covering the bones was removed and bones were treated with a 15% hydrogen peroxide solution (Sigma-Aldrich Inc., St. Louis, MO, USA) which was washed off with sterile saline after approximately 10–20 s. When the surface of the skull was clean and dry, a reference point for the entry point of the recording electrodes was marked (insular cortex (Ins): AP = −1.0 mm, ML = −6.1 mm; primary auditory cortex (AC): AP = −5.1 mm, ML = guided by landmarks on the lateral surface of parietal and temporal bones). Subsequently, 7 small holes for supporting screws were drilled and screws were tightly screwed into the frontal, parietal, and interparietal bones. Ground wire, soldered previously to one of the screws, was placed in the left frontal bone. The screws were fixed together and to the bone first with resin and then acrylic dental cements (Super-bond C&B, Sun Medical, Moriyama, Shiga, Japan; Coral-fix, Tel Aviv, Israel) forming a base of the implant. At the selected electrode implantation site, a thin polyimide tube was placed on the skull vertically and cemented with the rest of the implant base. The tube served as a guide to the implantation site for the next surgery. The free end of the ground wire was twisted and covered with a polyethylene cap cemented to the rest of the implant.

The wounds were cleaned and treated in situ with antibiotic ointment (synthomycine, chloramphenicol 3%, Rekah Pharm. Ind. Ltd., Holon, Israel). The skin was sutured in the anterior part of the implant with one or two sutures (Nylon, Assut sutures, Corgémont, Switzerland) to stretch the skin around the base of the implant. The skin around the wound was cleaned and covered with a povidone-iodine solution (10%). The rats received intraperitoneal injection of the antibiotic enrofloxacin 50mg/ml (5% W/V) in a dose of 15 mg/kg diluted with saline to 1 ml (Baytril, Bayer Animal Health GmbH, Leverkusen, Germany). After surgery, animals were housed individually to prevent them from chewing the implants. Carprofen or other similar NSAID dissolved in palatable wet food was provided at the home cage for the first few days after surgery. The rats were allowed at least 1 week of recovery post-surgery before restarting behavioral training.

#### Implantation of silicon probes

When the wounds were completely healed following the first surgery (14–28 days), the recording electrodes were implanted. To minimize tissue damage, a small craniotomy was made, drilling solely through the base of the implant, and thus leaving the healed skin intact to accelerate recovery and reduce the pain.

As previously, rats were initially anesthetized in an induction chamber with sevoflurane (8% in oxygen). After induction, rats were transferred to a stereotaxic instrument with a mask for gas anesthesia. Sevoflurane concentration was slowly adjusted to the level of 2–2.5% and maintained at this level throughout the procedure. The eyes were protected with sterile eye drops for dry eyes (Viscotears Liquid Gel, Carbomer: polyacrylic acid 2 mg/g, Berlin, Germany) and body temperature was controlled by a closed loop heating system with a rectal probe.

The dental cement above the implantation site marked by polyimide tube was removed gradually using a dental drill until the skull was exposed. The craniotomy was performed by drilling, and a 0.4–0.8-mm-long slit in the dura was gently resected. The electrodes were slowly inserted into the brain tissue using a single axis micromanipulator (MO-10, Narishige, Tokyo, Japan). The craniotomy was sealed with paraffin oil and elastic silicone polymer (Duragel, Cambridge Neurotech, UK). The microdrive and connector were fixed to the base of the implant with acrylic dental cement (see Additional file 24 for details). A ground wire was soldered between the base and the electrodes connector and covered with acrylic dental cement. To mechanically stabilize the connection between the implant and the recording device, an additional supporting connector was cemented to the implant (6 pins of 853 Interconnect Socket, MILL-MAX MFG. CORP., New York, USA). A custom plastic enclosure with a screw cap was cemented for implant protection. The robust design of the implant protected the microdrive and the electrodes against mechanical stress while the rats were moving, improving recording stability (Additional file 24d-f). The weight of the whole construct did not exceed 11.5 g.

At the end of the surgery, to prevent postoperative pain, rats received a subcutaneous injection of Carprofen 50mg/ml (12 mg/kg). Rats received intraperitoneal injection of enrofloxacin 50mg/ml (15 mg/kg) diluted with saline to 1 ml. Rats were allowed at least 3 days of recovery post-implantation before recordings.

### Wireless electrophysiology

Two wireless recording systems were used: (1) modular 64 channel neural logger (RatLog-64, Deuteron Technologies, Jerusalem, Israel) and (2) 64-channel wireless transmission system (TBSI W64, Triangle BioSystems International, Durham, NC, USA).

#### Neural logger

For the experiments described here, we used a single processor board and a single amplifier board to record from 32-channel silicon probes at a sampling rate of 32 kHz. The data were saved on a 64-GB microSD card, and copied to a computer after each session. The logger was equipped with an audio microphone and 9-axis motion sensor. An electrically insulated, compact enclosure for the logger, with a separate compartment for a battery, protected the logger from mechanical shocks (Additional file 24). The enclosure included an interconnector, to protect the board connector against mechanical damage caused by attaching and releasing over hundreds of recording sessions (Additional file 24a). The interconnector was also used to rotate the device about 34° backwards from the vertical axis, to allow the rats free movement and free access to the ports in the IAs (Additional file 24b). The enclosure of the logger had a supporting connector (6 pins of 852 + 853 Interconnect Socket, MILL-MAX MFG. CORP., New York, USA) that matched the supporting connector attached to the implant, in addition to a 36-pin Omnetics connector (A79029-001, Omnetics Connector Corporation, Minneapolis, MN, USA) for the electrical signals from the electrodes. The total weight of all elements was approximately 16 g. The load was balanced, to avoid pulling the rat head in any direction. Before every recording session, the device was additionally secured with autoclave sticky tape (Sigma-Aldrich, St. Louis, MO, USA) to prevent loosening of the connectors during the 2–3 h duration of the recording sessions. Maximal recording time using a 300-mAh battery was about 3 h (LiPo battery 582030).

#### Wireless analog transmission system

The system consisted of an analog transmitter and receiver (TBSI W64, Triangle BioSystems, Durham, NC, USA). The output signal of the receiver was routed to a data acquisition system (AlphaLab SnR, Alpha Omega, Additional file 1, item 15) and digitized at a sampling rate of 44 kHz. The recordings were made from 32-channel silicon probes as above. A small interconnector with a battery holder was prepared in the lab. The headstage was placed horizontally with a battery holder on its left side enabling animals to freely access to all locations in the experimental arena, and in particular to the IAs. The total weight of all elements was approximately 13.5 g of well-balanced load. Before each recording session, the device was additionally secured with autoclave sticky tape (Sigma-Aldrich, St. Louis, MO, USA) to prevent loosening of connectors contact during long active behavioral sessions (up to 12 h). Maximal recording time using a 260 mAh battery was about 11.5 h (LiPo battery 601240).

### Recordings

Neural signals were recorded in reference to a ground placed in the frontal bone. For the logger, the analog bandpass filter was set to 10–7000 Hz or 300–7000 Hz depending on the experiment. For the wireless system, the low frequency of the bandpass filter was set to 0.07 Hz. Recording sessions took place 5 days a week and implants were checked daily. Spiking activity was screened immediately after each recording session was finished. The electrodes were kept in the same position as long as spiking activity was detected on many contacts. When signals deteriorated, the animals were briefly sedated with sevoflurane and the electrodes were lowered in steps 25, 50, or 100 µm into the brain tissue. Electrodes were moved typically every 1–7 days. Electrodes were never moved up.

### Statistical analysis

Exploratory analysis of the dependence of spike trains on any of the measured parameters was conducted using linear mixed effects models (Matlab function fitlme). Explanatory variables included location and kinematic parameters, body and head direction parameters, and sound presentations. The models were fitted to the firing rates of the individual units collected in all experimental sessions of each rat. To check the effect of any of these parameters on the neuronal responses, it was used as a fixed effect (in order to account for a non-zero mean across the neuronal population) as well as a random factor depending on each recorded unit. These random factors are highly regularized (they are estimated under the assumption that they are instances of a Gaussian variable with a data-dependent covariance matrix [85]) and are therefore conservative estimates of the dependence of the firing rate of each specific unit on the parameter of interest.

We selected for further study units with prominent random effects. Prominent random effects were defined as effects whose magnitude was greater than the standard deviation of the corresponding fixed effect. The logic behind this choice is based on the observation that for a unit that has a prominent random effect, the dependence of its firing rate on the parameter of interest is different from that of the population mean. This method identified sound-sensitive neurons, but in addition it identified rat location, velocity, and head-body angle as variables that affected the activity of many units in all rats.

We highlight in this paper specifically location-sensitive units and head-body angle-sensitive units. We selected for display in the paper exemplary units that had prominent random effects, and then used 1-way ANOVA to confirm the significance of the dependences of firing rates on the variable of interest for these specific units. These tests are reported in the “Results” section. To increase our confidence in these results, we also performed permutation tests, for the units that showed location-dependent firing rates. Just permuting locations between time bins would have destroyed the dependencies between successive angles that are present in the data, and therefore we used the following approach for generating surrogate data for the permutation tests. First, we unwrapped the angles (matlab routine unwrap). We then performed phase randomization in the Fourier domain. The result was a surrogate time series of angles that kept the correlations of the original angles, but had a Gaussian distribution. We transformed that Gaussian distribution back to the original distribution of angles by using the empirical distribution functions: if we denote by *F* the empirical distribution function of the original angles (after unwrapping) and by *G* that of the Gaussian phase-randomized data *x(t)*, then final surrogate data used was *F(G*^*-1*^*(x(t)))*. This time series had the same distribution as the original time series of angles as well as the same second-order correlation structure. We repeated this procedure 100 times. For all location-sensitive neurons, the F of the ANOVA test for the surrogate data was smaller than that of the original data in all 100 repeats, so that *p*<0.01 in all cases.

While units could show sensitivity to multiple variables, we verified the independence of the effects of the variable of interest from all other variables. Thus, all examples shown here were not the result of a spurious correlation due to another primary dependence. We illustrate such tests with an example of location sensitivity. For each unit, the average firing rate as a joint function of location and one additional variable (for example, velocity) was computed, usually using a 10-by-10 grid of bins, and represented as a matrix. In order to check the independence of the tuning to the two variables, a rank 1 approximation was computed for this matrix using nonnegative matrix factorization (Matlab function nnmf, see Additional file 15). We then compared the matrix containing the average firing rates with its rank 1 approximation. To measure this, we divided the L2 norm of the residual by that of the original matrix. Small numbers represented good approximations and therefore a high level of independence. To follow standard usage, we subtracted this number from 1 and used that as the analog of the fraction of data variability explained by the rank 1 approximation.

Since the original matrix was estimated from a finite sample, it is expected to have a high rank even if its ideal structure has rank 1. Therefore, two methods were used to estimate the expected fraction of data variability explained by a rank 1 approximation to a noisy data matrix whose underlying structure is also of rank 1. In the first method the original data was used for bootstrapping: the firing rates observed within each location bin were resampled, and assigned to random velocity bins according to their observed probability. The mean for each one of the matrix entries was calculated. The resulting 10-by-10 matrix was processed in the same way as the original data. This process was repeated for 100 times, the fraction of explained variability averaged and compared to that of the original matrix. In the second method, the rank 1 approximation was used to generate surrogate data with Poisson distribution, using the same number of counts in each bin of the original matrix. This matrix was processed in the same way as the original data, and the fraction of explained data variability from this process was compared to that of the original data.

All the units were also tested for the effects of stimulus-driven responses (Additional files 14 and 16). The mean responses with and without sound presentations were computed as a function of the relevant parameter (in each location bin for the location-sensitive units, for each head-body angle bin for the head-body sensitive units). The mean firing rates in the presence and absence of sound were then compared using a paired *t*-test.

In all statistical analyses, significance was defined as *p*<0.05. Exact data are reported within the “Results” section and include the model, the statistic tested, and the *p*-value.

### Supplementary Information

**Additional file 1:** **Figure S1.** Scheme of hardware connections of Rat Interactive Foraging Facility (RIFF).

**Additional file 2:** **Figure S2.** Important dimensions in the RIFF. (A) Wall component dimensions, including those for walls with and without speakers, covers hiding foot shock grids, and vertical aluminum skeleton parts of the arena with grooves for wall placement. (B) Detailed dimensions of the speaker holder, an essential component of the experimental arena for providing well-controlled auditory stimuli.

**Additional file 3:** **Figure S3.** Structure of the two tasks described in the paper. (a) The multiple strategies task (St+). On the left, a flowchart of the experiment is displayed as a real-time loop. At the check position stage, three strategies were available to the rat, marked as A, B and C. These strategies are illustrated in the diagram of the arena on the right, using the same color code. The A strategy consisted of moving from one interaction area to another (usually a neighboring area), which was then selected as the next target. The B strategy consisted of cycling from an A area to the associated B area, which led to the selection of the same A area as the next target. The C strategy consisted of moving to a C area, in which case a random port was selected as the next target. (b) Diagram of the localization/discrimination task (L/D). Flowchart of the experiment real-time loop (left) and the corresponding events in a diagram of the arena (right), plotted with the same color code.

**Additional file 4:** **Figure S4.** Feature extraction from the video images. (a) A feed-forward convolutional neural network estimates the locations of the head, the base of the neck and the base of the tail for each input image. These three markers are then used for the calculation of the body and head angles (bottom right image). (b) The table details the custom architecture of the neural network, which is optimized for the grayscale rectangular input, reducing the number of parameters of the trained model and decreasing the inference times.

**Additional file 5:** **Video S1.** Automatic detection of 3 rat body points. An artificial neural network automatically marks the locations of the nose, base of the neck and the tail on a rat image (yellow, red and blue dots, respectively).

**Additional file 6:** **Video S2.** Calculation of the body direction of therat. Green arrow indicates the calculated body direction from the base of the tail towards the base of the neck (blue and yellow points from Additional file 5).

**Additional file 7:** **Figure S5.** Neural data processing and spike sorting. (a) Extracellular neural recordings in freely behaving rats (32 simultaneously recorded channels) include periods of high noise (left panel). Noise components that are common to all channels can be largely removed by subtracting the average waveform (middle panel). The remaining noise segments are identified by their amplitude and by their high variance across channels, and are zeroed (right panel). The resulting neural data is then processed by Kilosort2. (b) Implausible spike shapes in the clusters detected by Kilosort2 are automatically detected. The top example was automatically identified as a spike, while the bottom two examples were identified as noise. (c) Statistics of the neural activity are produced for each cluster and used for manual classification.

**Additional file 8:** **Video S3.** Rat tracking module. The location of the rat is determined in each video frame, at 30 FPS, as it freely moves inside the arena (green asterisk marks the center of mass of the rat). The location information is transmitted to the main control computer, while the video frames are stored and used to extract additional behavioral features during the post-processing.

**Additional file 9:** **Video S4.** Exemplary behavior of an expert rat in the St+ task at its full complexity. Rat successfully switches between zones A1 and B6, avoids the air puff in response to the warning sound, and after the safe sound, it continues to use A and B zones while missing an opportunity coming from the C zone (see Additional file 3a). The St+ task provides substantial freedom to the rats, allowing them to optimize their behavior with respect to their individual preferences for size and type of rewards while effectively avoiding the punishment. Red circles mark active speakers; yellow circle indicates detected nose-poke; green circle indicates dispensed reward. White arrows indicate automatically extracted head and body directions. Dots behind the rat indicate previous location, color indicates speed (blue - slowest, red - fastest). The legend in the top right corner indicates the area where the rat is located. The name of the target sound appears while it plays.

**Additional file 10:** **Video S5.** Exemplary behavior of an expert rat in the L/D task. Rat goes to the center zone of the arena to initiate a trial with a sound presentation (see Additional file 3b and “Methods” for a full description of the task). All markers are the same as in Additional file 9.

**Additional file 11:** **Figure S6.** Changes in target arrival time distributions from Day 1 to Day 2. Shown are log likelihoods of target arrival time in 1 second bins, for Day 2 compared to Day 1. Only rewarded trials are included. Each bar depicts log10 of the probability to arrive at the target in this time bin on Day 2, relative to Day 1. The attention sound is denoted by a black line, and the target sound is denoted by a green line. The number of rewarded trials on each day is denoted in the figure (N1, N2). In each rat, late target arrivals are less likely on Day 2 than on Day 1, and in each rat except rat 5, target arrivals before the attention sound are more likely on Day 2 than on Day 1. The distributions were significantly different in each rat (two-sample Kolmogorov-Smirnov test; *P* < 0.01 in each rat; *P* = 4.62 × 10^-15^ for all rats). In all rats except rat 5, target arrival times on Day 2 were significantly earlier than on Day 1 (two-sample t-test; *P* < 0.006 in rats 4, 6, 7, 8; *P* = 0.13 in rat 5; *P* = 1.13 × 10^-26^ for all rats). These data indicate that four out of five rats were more likely to arrive in the target area earlier on Day  2 compared to Day 1 while one rat (rat 5) increased the probability to arrive at the target area between the attention and target sounds relative to earlier and later times. Thus, all rats modified their behavioral strategies in day 2 relative to day 1 in order to better conform to the contingencies of the task. Compare also Fig. 4d in the main text.

**Additional file 12:** **Figure S7.** Classification of trials into three types. (a) Angular running speed for all trials performed by rat 4 on day 1. For each trial, the maximal angular speed in the clockwise and counterclockwise direction was extracted in a time window lasting from 0.5 s to 9 s following the feedback sound of the previous trial. A trial was classified as "Sit" (gray lines) if the absolute value of the angular speed never exceeded 0.25 radians/s (14.3 degrees/s), otherwise as "Run clockwise" (blue lines) or "Run counterclockwise" (red lines), according to the direction with the higher maximal speed. (b) Trial clusters were clearly separated in all rats. Scatter plots show each trial of each rat and each day according to the maximal angular speed in the clockwise and counterclockwise directions. Colors as in (a). The number of trials of each trial cluster are indicated.

**Additional file 13:** **Figure S8.** High-resolution responses of the units in Figs. 5e-h. Response of the units is depicted as probability of firing as a function of the radial location. Each point reflects the weighted average of the firing rates in nearby radial locations. The weights were calculated using a Gaussian window with a standard deviation of 1o. The gray area is standard deviation. In each plot, the maximal response was shifted to 0o.

**Additional file 14:** **Figure S9.** Joint sensitivity of the units in Figs.5e-h to location and sound. (a) Mean responses in the presence (blue) and absence (red) of sound. Error barsare s.e.m. (b) Scatter plots of the data as in (a). Horizontal error bar indicates s.e.m during sound presentation, vertical error bar indicates s.e.m during silence. The number of instances from which the mean and s.e.m were derived are indicated on the right of each point (during sound presentation), and on top (during silence). In case no sound presentations occurred in a location bin, the data is plotted on the y axis.

**Additional file 15:** **Figure S10.** Joint sensitivity of the units in Figs.5e-h to location and head-body angle or velocity. (a) Mean firing rates for location (abscissa) and relative head-body angle (ordinate). (b) Non-negative rank 1 matrix approximation of the matrices in (a). Top right corner: the fraction of the data variability explained by the rank 1 approximation for the original data / bootstrapping method / Poisson distribution approximation (see "Methods" for details). (c) Mean firing rates for location (abscissa) and velocity (ordinate). (d) Same as (b) for the matrices in (c).

**Additional file 16:** **Figure S11.** Joint sensitivity of the units in Figs.5i-l to location and sound. Same format as Additional file 14.

**Additional file 17:** **Figure S12.** Joint sensitivity of the units in Figs.5i-l to head-body angle and absolute head angle, absolute body angle, or location. Same representations as in Additional file 15. The abscissa represents head-body angles, while the ordinate represents absolute head angle ((a) and (b)), absolute body angle ((c)and (d)), and location ((e) and (f)).

**Additional file 18:** **Figure S13.** The real-time imaging module. (a) Diagram of the camera (DMK 33G445 GigE, TheImagingSource) and the light ring, mounted on the ceiling above the arena’s center. (b) Synchronization diagram of the image stream. Triggers that indicate frame acquisition were sent to a digital processor that sub-sampled them from 30 Hz to 1 Hz. The 1 Hz triggers were then simultaneously recorded on the common synchronization hardware, and also powered a LED in the field of view of the camera. (c) Graphical user interface of the real-time imaging module. The LED is marked by a small red square on the right side of the arena. The rat center of mass is marked by a green asterisk. (d) For efficient data storage, rat images were cropped around its center of mass and stored in a multi-page .tif file. A cropped image of the LED was stored in the upper left corner of each image, allowing for time synchronization during the post-processing steps.

**Additional file 19:** **Figure S14.** Analysis of the timing behavior of the main control loop. A. Histogram of running times of the main loop on the master control computer, when no delays were imposed by waiting to external hardware. B-f. Histogram of latencies between image acquisition by the camera and various events on the video processing computer (b and c) and the master control computer (d-f).

**Additional file 20:** **Figure S15.** Exemplary frames of the pose estimation algorithm. The CNN model was trained in a supervised manner to predict the nose, neck and the tail of the rat (blue, orange and green circles, respectively) in 1500 images that were manually tagged (red crosses).

**Additional file 21:** **Video S6.** Distance of the estimated pose features from the manual tags. The model estimates the pose features (head, neck and tail) with precision of ~0.65cm (see Additional file 22). This allows for exact calculation of the body and the head directions (right plot).

**Additional file 22:** **Figure S16.** Accuracy of the pose estimation model. The precision of the model was estimated by calculating the euclidean distance from the predicted points to the manualtags. The mean error for the head, the neck and the tail points was 0.62 cm, 0.66 cm and 0.62 cm, respectively.

**Additional file 23:** **Video S7.** Experiment visualizer. We provide a custom visualizer for recorded experiments that can be used to show the rat trajectory and nose-pokes, sounds played, and neural activity. The experiment is replayed at 30 frames per second. The video shows how to load an experiment into the visualizer, and how to use many of its functions.

**Additional file 24:** **Figure S17.** An approach for chronic wireless recordings in rats. (a) The neural logger and battery are in the protective plastic case that can be attached to the electrode’s connector. The 300 mAh battery is placed in a separate compartment, connects to the logger through a micro-JST connector, and can be easily changed during the experiment. Anti-static foam pads are placed on the sides of the logger components (amplifier and processor boards) to protect the logger against mechanical shocks. The protective case has a 36-pin omnetics connector matching that on the 32-channel silicon probe, as well as a small Mill-Max connector which mechanically stabilizes the case during recording sessions. The silicon probe is mounted on the Microdrive. (b and c) Side and front views of the recording set. The device is inclined to the back in order to allow rats natural movements and undisturbed access to ports. (d) The silicon probe is mounted on the Microdrive cemented to the skull. The moveable parts of the implant are covered with paraffin oil. The flex cable of the probe is bent to provide a long travel distance for the electrodes. (e) Finished implant with protective enclosure. (f) Female rats with a 32-channel moveable silicon probe implant and the wireless data logger in the case with a battery. The recording set enables natural movements, is easily carried by the rats, and is well protected against mechanical shocks. The enclosure can be closed with a plastic cap (orange) to protect the implant in the home cage.

## Data Availability

An online resource containing (1) the data and code to create the main figures in this article, (2) the code that operates the rat tracker and the RIFF and runs the St+ and L/D experiments, (3) the data analysis pipeline that transforms the collected raw data into files for further analysis, (4) the experiment visualizer to replay experiments from their recorded data, (5) an extensive wiki with detailed information on all code and software related to the RIFF is available online as follows: the main repository is available at https://github.com/jniediek/RIFF_publication, and the wiki can be found at https://github.com/jniediek/RIFF_publication/wiki. The full data from two sample experiment sessions are provided. A citable online resource of one sample session is available on figshare (https://doi.org/10.6084/m9.figshare.15082971.v1).

## Abbreviations

AC: Primary auditory field
IA: Interaction area
Ins: Posterior insular cortex
IQR: Interquartile range
L/D: Localization/discrimination task
LME: Linear mixed effects
MDP: Markov Decision Process
RIFF: Rat Interactive Foraging Facility
St+: Multiple-strategy task

## References

[1] Schnupp J, Nelken I, King AJ (2010). Auditory neuroscience: making sense of sound.

[2] Shadmehr R, Smith MA, Krakauer JW (2010). Error correction, sensory prediction, and adaptation in motor control. Annu Rev Neurosci..

[3] Carandini M, Churchland AK (2013). Probing perceptual decisions in rodents. Nat Neurosci..

[4] Poo M-M, Pignatelli M, Ryan TJ, Tonegawa S, Bonhoeffer T, Martin KC (2016). What is memory? The present state of the engram. BMC Biol..

[5] Saraf-Sinik I, Assa E, Ahissar E (2015). Motion makes sense: an adaptive motor-sensory strategy underlies the perception of object location in rats. J Neurosci..

[6] Musall S, Kaufman MT, Juavinett AL, Gluf S, Churchland AK (2019). Single-trial neural dynamics are dominated by richly varied movements. Nat Neurosci.

[7] Sutton RS, Barto AG (2018). Reinforcement learning: an introduction.

[8] Mallory CS, Hardcastle K, Campbell MG, Attinger A, Low IIC, Raymond JL (2021). Mouse entorhinal cortex encodes a diverse repertoire of self-motion signals. Nat Commun.

[9] Pachitariu M, Sridhar S, Stringer C. Solving the spike sorting problem with Kilosort. bioRxiv. 2023. 10.1101/2023.01.07.523036.

[10] Pachitariu M. Kilosort2, https://github.com/MouseLand/Kilosort (2020).

[11] Jankowski MM, Polterovich A, Kazakov A, Niediek J, Nelken I. RIFF Software v1.0.1. zenodo. 2023. 10.5281/zenodo.8089123.

[12] Rodgers KM, Benison AM, Klein A, Barth DS (2008). Auditory, somatosensory, and multisensory insular cortex in the rat. Cereb Cortex.

[13] Kimura A, Imbe H, Donishi T (2010). Efferent connections of an auditory area in the caudal insular cortex of the rat: anatomical nodes for cortical streams of auditory processing and cross-modal sensory interactions. Neuroscience.

[14] Sofroniew NJ, Cohen JD, Lee AK, Svoboda K (2014). Natural whisker-guided behavior by head-fixed mice in tactile virtual reality. J Neurosci.

[15] Keller GB, Bonhoeffer T, Hübener M (2012). Sensorimotor mismatch signals in primary visual cortex of the behaving mouse. Neuron.

[16] Harvey CD, Collman F, Dombeck DA, Tank DW (2009). Intracellular dynamics of hippocampal place cells during virtual navigation. Nature.

[17] Hölscher C, Schnee A, Dahmen H, Setia L, Mallot HA (2005). Rats are able to navigate in virtual environments. J Exp Biol.

[18] Aghajan ZM, Acharya L, Moore JJ, Cushman JD, Vuong C, Mehta MR (2015). Impaired spatial selectivity and intact phase precession in two-dimensional virtual reality. Nat Neurosci.

[19] Go MA, Rogers J, Gava GP, Davey CE, Prado S, Liu Y, et al. Place cells in head-fixed mice navigating a floating real-world environment. Front Cell Neurosci. 2021;15. 10.3389/fncel.2021.618658. 10.3389/fncel.2021.618658 PMC7906988 33642996

[20] Minderer M, Harvey CD, Donato F, Moser EI (2016). Virtual reality explored. Nature.

[21] Radvansky BA, Dombeck DA (2018). An olfactory virtual reality system for mice. Nat Commun.

[22] Dombeck DA, Harvey CD, Tian L, Looger LL, Tank DW (2010). Functional imaging of hippocampal place cells at cellular resolution during virtual navigation. Nat Neurosci.

[23] Dombeck DA, Khabbaz AN, Collman F, Adelman TL, Tank DW (2007). Imaging large-scale neural activity with cellular resolution in awake, mobile mice. Neuron.

[24] Bjerre A-S, Palmer LM. Probing cortical activity during head-fixed behavior. Front Mol Neurosci. 2020;13:30. 10.3389/fnmol.2020.00030 PMC7059801 32180705

[25] Huang K-H, Rupprecht P, Frank T, Kawakami K, Bouwmeester T, Friedrich RW (2020). A virtual reality system to analyze neural activity and behavior in adult zebrafish. Nat Methods.

[26] Whishaw IQ, Faraji J, Kuntz J, Mirza Agha B, Patel M, Metz GAS (2017). Organization of the reach and grasp in head-fixed vs freely-moving mice provides support for multiple motor channel theory of neocortical organization. Exp Brain Res.

[27] Ravassard P, Kees A, Willers B, Ho D, Aharoni D, Cushman J (2013). Multisensory control of hippocampal spatiotemporal selectivity. Science.

[28] Keating P, Dahmen JC, King AJ (2013). Context-specific reweighting of auditory spatial cues following altered experience during development. Curr Biol.

[29] Tsoar A, Nathan R, Bartan Y, Vyssotski A, Dell’Omo G, Ulanovsky N (2011). Large-scale navigational map in a mammal. PNAS.

[30] Khan Z, Herman RA, Wallen K, Balch T (2005). An outdoor 3-D visual tracking system for the study of spatial navigation and memory in rhesus monkeys. Behav Res Methods.

[31] Fry SN, Bichsel M, Müller P, Robert D (2000). Tracking of flying insects using pan-tilt cameras. J Neurosci Methods.

[32] Lim J, Celikel T (2019). Real-time contextual feedback for close-loop control of navigation. J Neural Eng.

[33] Ballesta S, Reymond G, Pozzobon M, Duhamel J-R (2014). A real-time 3D video tracking system for monitoring primate groups. J Neurosci Methods.

[34] Matsumoto J, Urakawa S, Takamura Y, Malcher-Lopes R, Hori E, Tomaz C (2013). A 3D-video-based computerized analysis of social and sexual interactions in rats. PLOS One.

[35] de Chaumont F, Coura RD-S, Serreau P, Cressant A, Chabout J, Granon S (2012). Computerized video analysis of social interactions in mice. Nat Methods.

[36] de Chaumont F, Ey E, Torquet N, Lagache T, Dallongeville S, Imbert A (2019). Real-time analysis of the behaviour of groups of mice via a depth-sensing camera and machine learning. Nat Biomed Eng.

[37] Weissbrod A, Shapiro A, Vasserman G, Edry L, Dayan M, Yitzhaky A (2013). Automated long-term tracking and social behavioural phenotyping of animal colonies within a semi-natural environment. Nature Communications..

[38] Pérez-Escudero A, Vicente-Page J, Hinz RC, Arganda S, de Polavieja GG (2014). idTracker: tracking individuals in a group by automatic identification of unmarked animals. Nat Methods.

[39] Romero-Ferrero F, Bergomi MG, Hinz RC, Heras FJH, de Polavieja GG (2019). idtracker.ai: tracking all individuals in small or large collectives of unmarked animals. Nat Methods.

[40] Straw AD, Branson K, Neumann TR, Dickinson MH (2011). Multi-camera real-time three-dimensional tracking of multiple flying animals. J Royal Soc Interface..

[41] Hong W, Kennedy A, Burgos-Artizzu XP, Zelikowsky M, Navonne SG, Perona P (2015). Automated measurement of mouse social behaviors using depth sensing, video tracking, and machine learning. PNAS.

[42] Shemesh Y, Sztainberg Y, Forkosh O, Shlapobersky T, Chen A, Schneidman E (2013). High-order social interactions in groups of mice. eLife..

[43] Aguillon-Rodriguez V, Angelaki D, Bayer H, Bonacchi N, Carandini M, The International Brain Laboratory (2021). Standardized and reproducible measurement of decision-making in mice. Life.

[44] Yartsev MM, Ulanovsky N (2013). Representation of three-dimensional space in the hippocampus of flying bats. Science.

[45] Finkelstein A, Derdikman D, Rubin A, Foerster JN, Las L, Ulanovsky N (2015). Three-dimensional head-direction coding in the bat brain. Nature.

[46] Ginosar G, Aljadeff J, Burak Y, Sompolinsky H, Las L, Ulanovsky N (2021). Locally ordered representation of 3D space in the entorhinal cortex. Nature.

[47] Nourizonoz A, Zimmermann R, Ho CLA, Pellat S, Ormen Y, Prévost-Solié C (2020). EthoLoop: automated closed-loop neuroethology in naturalistic environments. Nat Methods.

[48] Adamsky A, Kol A, Kreisel T, Doron A, Ozeri-Engelhard N, Melcer T (2018). Astrocytic activation generates de novo neuronal potentiation and memory enhancement. Cell.

[49] Ressler RL, Goode TD, Kim S, Ramanathan KR, Maren S (2021). Covert capture and attenuation of a hippocampus-dependent fear memory. Nat Neurosci.

[50] Ross TW, Easton A (2022). Rats use strategies to make object choices in spontaneous object recognition tasks. Sci Rep.

[51] Pereira TD, Tabris N, Matsliah A, Turner DM, Li J, Ravindranath S (2022). SLEAP: a deep learning system for multi-animal pose tracking. Nat Methods.

[52] Schweihoff JF, Loshakov M, Pavlova I, Kück L, Ewell LA, Schwarz MK (2021). DeepLabStream enables closed-loop behavioral experiments using deep learning-based markerless, real-time posture detection. Commun Biol.

[53] Mathis A, Mamidanna P, Cury KM, Abe T, Murthy VN, Mathis MW (2018). DeepLabCut: markerless pose estimation of user-defined body parts with deep learning. Nat Neurosci.

[54] Kabra M, Robie AA, Rivera-Alba M, Branson S, Branson K (2013). JAABA: interactive machine learning for automatic annotation of animal behavior. Nat Methods.

[55] Hsu AI, Yttri EA (2021). B-SOiD, an open-source unsupervised algorithm for identification and fast prediction of behaviors. Nat Commun.

[56] Berman GJ, Choi DM, Bialek W, Shaevitz JW (2014). Mapping the stereotyped behaviour of freely moving fruit flies. J R Soc Interface.

[57] Marshall JD, Aldarondo DE, Dunn TW, Wang WL, Berman GJ, Ölveczky BP (2021). Continuous whole-body 3D kinematic recordings across the rodent behavioral repertoire. Neuron.

[58] Gardner RJ, Hermansen E, Pachitariu M, Burak Y, Baas NA, Dunn BA (2022). Toroidal topology of population activity in grid cells. Nature.

[59] Bruinsma B, Terra H, de Kloet SF, Luchicchi A, Timmerman AJ, Remmelink E (2019). An automated home-cage-based 5-choice serial reaction time task for rapid assessment of attention and impulsivity in rats. Psychopharmacology (Berl).

[60] Fizet J, Cassel J-C, Kelche C, Meunier H (2016). A review of the 5-Choice Serial Reaction Time (5-CSRT) task in different vertebrate models. Neurosci Biobehav Rev.

[61] Toschi C, Hervig ME-S, Moazen P, Parker MG, Dalley JW, Gether U (2021). Adaptive aspects of impulsivity and interactions with effects of catecholaminergic agents in the 5-choice serial reaction time task: implications for ADHD. Psychopharmacology (Berl)..

[62] Steinmetz NA, Aydin C, Lebedeva A, Okun M, Pachitariu M, Bauza M, et al. Neuropixels 2.0: A miniaturized high-density probe for stable, long-term brain recordings. Science. 2021;372:eabf4588. 10.1126/science.abf4588 PMC8244810 33859006

[63] Poddar R, Kawai R, Ölveczky BP (2013). A fully automated high-throughput training system for rodents. PLOS One.

[64] Miller KJ, Botvinick MM, Brody CD (2017). Dorsal hippocampus contributes to model-based planning. Nat Neurosci..

[65] Gronskaya E, von der Behrens W (2019). Evoked response strength in primary auditory cortex predicts performance in a spectro-spatial discrimination task in rats. J Neurosci.

[66] de Hoz L, Nelken I (2014). Frequency tuning in the behaving mouse: different bandwidths for discrimination and generalization. PLoS One..

[67] Rosenberg M, Zhang T, Perona P, Meister M (2021). Mice in a labyrinth show rapid learning, sudden insight, and efficient exploration. Elife..

[68] Takemoto M, Hasegawa K, Nishimura M, Song W-J (2014). The insular auditory field receives input from the lemniscal subdivision of the auditory thalamus in mice. J Comp Neurol.

[69] Jankowski MM, O’Mara SM (2015). Dynamics of place, boundary and object encoding in rat anterior claustrum. Front Behav Neurosci.

[70] Jankowski MM, Passecker J, Islam MN, Vann S, Erichsen JT, Aggleton JP (2015). Evidence for spatially-responsive neurons in the rostral thalamus. Front Behav Neurosci.

[71] Long X, Deng B, Cai J, Chen ZS, Zhang S-J. A compact spatial map in V2 visual cortex. bioRxiv. 2021. 10.1101/2021.02.11.430687.

[72] Long X, Zhang S-J (2021). A novel somatosensory spatial navigation system outside the hippocampal formation. Cell Res.

[73] Mao D, Kandler S, McNaughton BL, Bonin V (2017). Sparse orthogonal population representation of spatial context in the retrosplenial cortex. Nat Commun.

[74] Yin A, Tseng PH, Rajangam S, Lebedev MA, Nicolelis MAL (2018). Place cell-like activity in the primary sensorimotor and premotor cortex during monkey whole-body navigation. Sci Rep.

[75] Lopes G, Bonacchi N, Frazão J, Neto JP, Atallah BV, Soares S (2015). Bonsai: an event-based framework for processing and controlling data streams. Front Neuroinform.

[76] Buccino AP, Lepperød ME, Dragly S-A, Häfliger P, Fyhn M, Hafting T (2018). Open source modules for tracking animal behavior and closed-loop stimulation based on Open Ephys and Bonsai. J Neural Eng.

[77] Akam T, Lustig A, Rowland JM, Kapanaiah SK, Esteve-Agraz J, Panniello M (2022). Open-source, Python-based, hardware and software for controlling behavioural neuroscience experiments. Elife..

[78] Amaro D, Ferreiro DN, Grothe B, Pecka M (2021). Source identity shapes spatial preference in primary auditory cortex during active navigation. Curr Biol..

[79] Saldeitis K, Happel MFK, Ohl FW, Scheich H, Budinger E (2014). Anatomy of the auditory thalamocortical system in the Mongolian gerbil: nuclear origins and cortical field-, layer-, and frequency-specificities. J Comp Neurol..

[80] Huet A, Batrel C, Tang Y, Desmadryl G, Wang J, Puel J-L (2016). Sound coding in the auditory nerve of gerbils. Hear Res.

[81] Kawahara H, Morise M, Takahashi T, Nisimura R, Irino T, Banno H (2008). Tandem-STRAIGHT: A temporally stable power spectral representation for periodic signals and applications to interference-free spectrum, F0, and aperiodicity estimation. 2008 IEEE International Conference on Acoustics, Speech and Signal Processing.

[82] Kazakov A, Jankowski MM, Nelken I (2018). Acoustic recordings data from an echoic environment and a toolkit for its analysis. Data Brief.

[83] He K, Zhang X, Ren S, Sun J. Delving deep into rectifiers: surpassing human-level performance on ImageNet Classification. arXiv. 2015 :150201852 [cs].

[84] Kingma DP, Ba J. Adam: a method for stochastic optimization. arXiv:14126980 [cs]. 2017.

[85] Verbeke G, Molenberghs G, editors. A model for longitudinal data. In: Linear Mixed Models for Longitudinal Data. New York: Springer; 2000. 19–29.

