# Supplementary material for: An automated, low-latency environment for studying the neural basis of behavior in freely moving rats
Source: BMC Biol. 2023 Aug 11;21:172. doi: 10.1186/s12915-023-01660-9 (PMC10416379; doi:10.1186/s12915-023-01660-9)
Supplement: Supplementary file 1 — Additional file 1: Figure S1. Scheme of hardware connections of Rat Interactive Foraging Facility (RIFF). [file 12915_2023_1660_MOESM1_ESM.pdf]

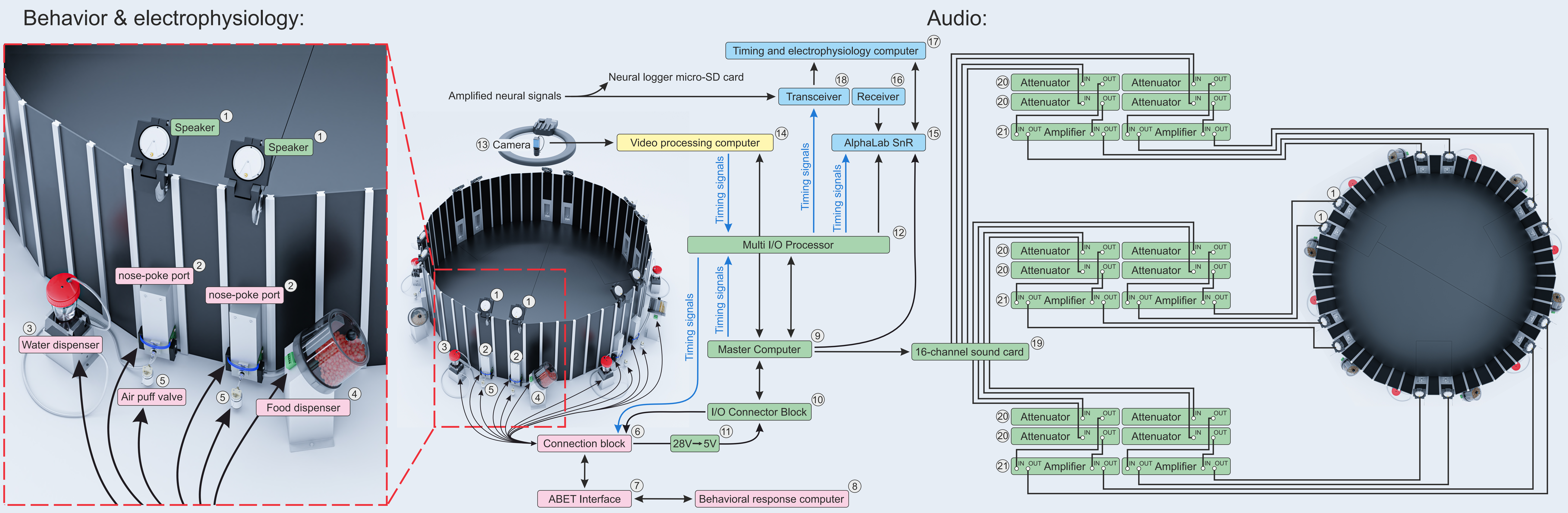

Additional file 1: Scheme of hardware connections of Rat Interactive Foraging Facility (RIFF). The information about the state of the environment is conveyed to the rats by speakers ① (MF1 Multi-Field Magnetic Speakers, TDT). In response to sounds, rats interact with nose-poke ports equipped with photo beams ② (SPECIAL.090-SE v1.0, LaFayette-Campden Instruments). Nose-poke ports also serve as a reward delivery area provided by fluid pump ③ (Model 80204, LaFayette-Campden Instruments) or precise food dispenser for 45 mg food tablets ④ (Model 80209, LaFayette-Campden Instruments). Each port is additionally equipped with an air-puff outlet controlled by a solenoid valve ⑤ (Series 3 miniature inert liquid valve, Parker). The photo beams of the nose-poke port, the reward dispensers, and the air puff valves are wired to connection block ⑥ (Model 81408, ABET Full I/O Module Connection Block, LaFayette-Campden Instruments). The connection block ⑥ is wired through two standard DB-25 Cables with ABET Interface ⑦ (Model 81501, ABET 2G Starter/Expansion Interface, LaFayette-Campden Instruments), which connects with behavioral response computer ⑧ through a PCI card (Model 81504e, ABET 2G PCIe Interface Card and Cable, LaFayette-Campden Instruments). The behavioral system is controlled through ABET software (Model 89501, ABET II Software for Operant Control, LaFayette-Campden Instruments) installed on behavioral response computer ⑧. Moreover, the master computer ⑨, which controls the experiments with custom matlab programs, can directly control some elements of the behavioral system through a digital IO card (PCI-DIO-96, National Instruments) connected to a shielded I/O Connector Block ⑩ (SCB-100, National Instruments). The hardware of the behavioral system operates with 28V signals. Therefore, inputs from connection block ⑥ to the I/O Connector Block ⑩ interfacing with the master computer ⑨ pass through custom-built voltage regulators 28V → 5V ⑪. Time triggers to the behavioral system are sent through Multi-I/O Processor ⑫ (RX8, TDT) wired to the connection block ⑥.

The behavioral system responds to the rat's position in the environment monitored by the camera ⑬ (Model DFK 23G445.I, The Imaging Source). The camera ⑬ is connected through a standard PCI video interface card to the video processing computer ⑭, which sends the information about the position of the rat to the master computer ⑨ on a dedicated ethernet line. The video processing computer ⑭ sends timing signals to the Multi-I/O Processor ⑫. The Multi-I/O Processor ⑫ receives all timing signals (sounds, camera, experiment state, and behavioral system) and channels them to AlphaLab SNR recording system ⑮ (Alpha Omega).

The electrophysiological signals were continuously recorded with a wireless analog transmission system with its receiver ⑯ (64-channel, Triangle BioSystems, Inc.) coupled with ALphaLab SnR recording system ⑮. The data is saved on the electrophysiology computer ⑰, or with neural logger communicating with the transceiver ⑱ (Deuteron Technologies) which receives timing signals from Multi-I/O Processor ⑫. In the latter option, data with time stamps were saved on a micro-SD card of the logger.

The audio files are stored on the master computer ⑨ and sent to the external 16-channel digital to analog converter ⑲ (M-16 and MADIface USB, RME Audio Interfaces). The level of the analog audio signals is set by programmable attenuators ⑳ (TDT, PA5), and the signal is amplified by stereo amplifiers ㉑ (TDT, SA1). Each stereo amplifier drives a pair of speakers ① of one interactive area.
