# Supplementary material for: An automated, low-latency environment for studying the neural basis of behavior in freely moving rats
Source: BMC Biol. 2023 Aug 11;21:172. doi: 10.1186/s12915-023-01660-9 (PMC10416379; doi:10.1186/s12915-023-01660-9)
Supplement: Supplementary file 2 — Additional file 2: Figure S2. Important dimensions in the RIFF. (A) Wall component dimensions, including those for walls with and without speakers, covers hiding foot shock grids, and vertical aluminum skeleton parts of the arena with grooves for wall placement. (B) Detailed dimensions of the speaker holder, an essential component of the experimental arena for providing well-controlled auditory stimuli. [file 12915_2023_1660_MOESM2_ESM.pdf]

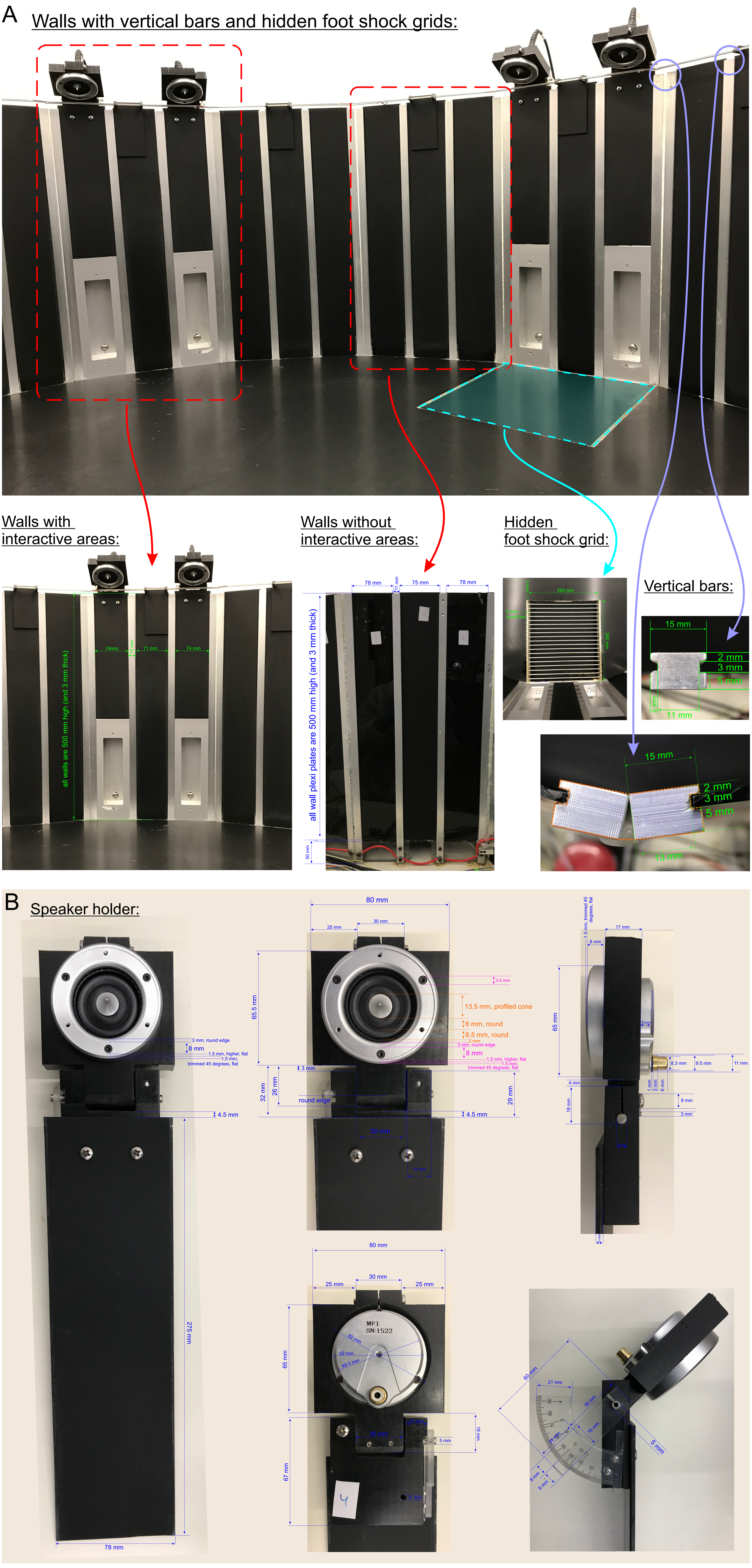

A
Walls with vertical bars and hidden foot shock grids:
Walls with interactive areas:
Walls without interactive areas:
Hidden foot shock grid:
Vertical bars:
all walls are 500 mm high (and 3 mm thick)
74mm
15 mm
71 mm
74 mm
78 mm
75 mm
78 mm
all wall plexi plates are 500 mm high (and 3 mm thick)
50 mm
260 mm
280 mm
15 mm
2 mm
3 mm
5 mm
11 mm
15 mm
2 mm
3 mm
5 mm
13 mm
B
Speaker holder:
3 mm, round edge
8 mm
1.5 mm, higher, flat
1.5 mm,
trimmed 45 degrees, flat
4.5 mm
275 mm
78 mm
80 mm
25 mm
30 mm
65.5 mm
3.5 mm
13.5 mm, profiled cone
6 mm, round
6.5 mm, round
3 mm, round edge
8 mm
1.5 mm, higher, flat
trimmed 45 degrees, flat
3 mm
32 mm
26 mm
round edge
29 mm
4.5 mm
17 mm
8 mm
65 mm
8.3 mm
9.5 mm
11 mm
4 mm
18 mm
8 mm
3 mm
80 mm
25 mm
30 mm
25 mm
65 mm
MF1
SN:1522
5 mm
18 mm
67 mm
60 mm
21 mm
5 mm
