# Supplementary figures and images for: An automated, low-latency environment for studying the neural basis of behavior in freely moving rats
Source: BMC Biol. 2023 Aug 11;21:172. doi: 10.1186/s12915-023-01660-9 (PMC10416379; doi:10.1186/s12915-023-01660-9)

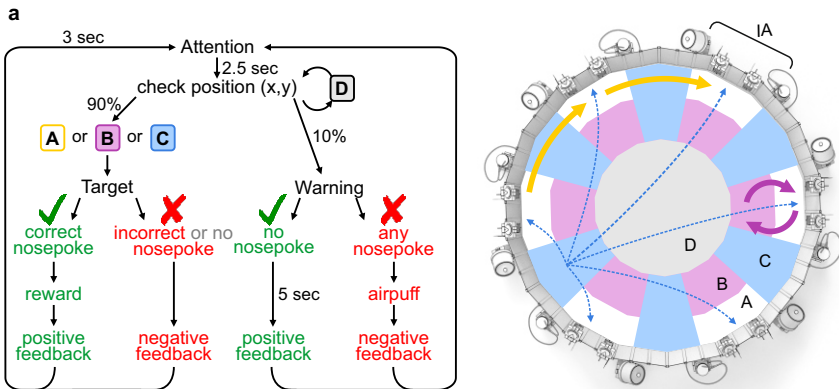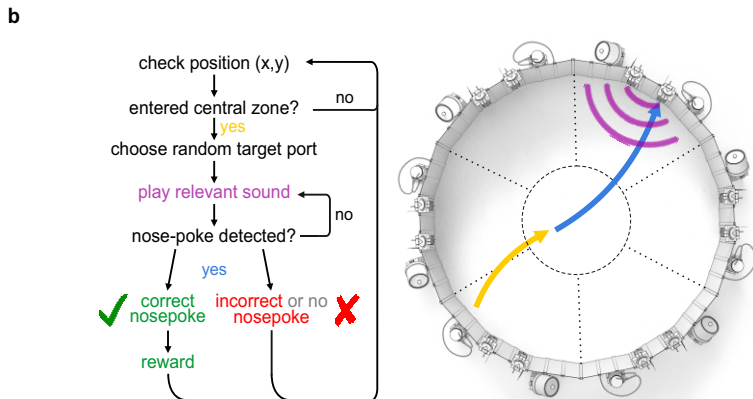

Supplement: Supplementary file 3 — Additional file 3: Figure S3. Structure of the two tasks described in the paper. (a) The multiple strategies task (St+). On the left, a flowchart of the experiment is displayed as a real-time loop. At the check position stage, three strategies were available to the rat, marked as A, B and C. These strategies are illustrated in the diagram of the arena on the right, using the same color code. The A strategy consisted of moving from one interaction area to another (usually a neighboring area), which was then selected as the next target. The B strategy consisted of cycling from an A area to the associated B area, which led to the selection of the same A area as the next target. The C strategy consisted of moving to a C area, in which case a random port was selected as the next target. (b) Diagram of the localization/discrimination task (L/D). Flowchart of the experiment real-time loop (left) and the corresponding events in a diagram of the arena (right), plotted with the same color code. [file 12915_2023_1660_MOESM3_ESM.pdf]

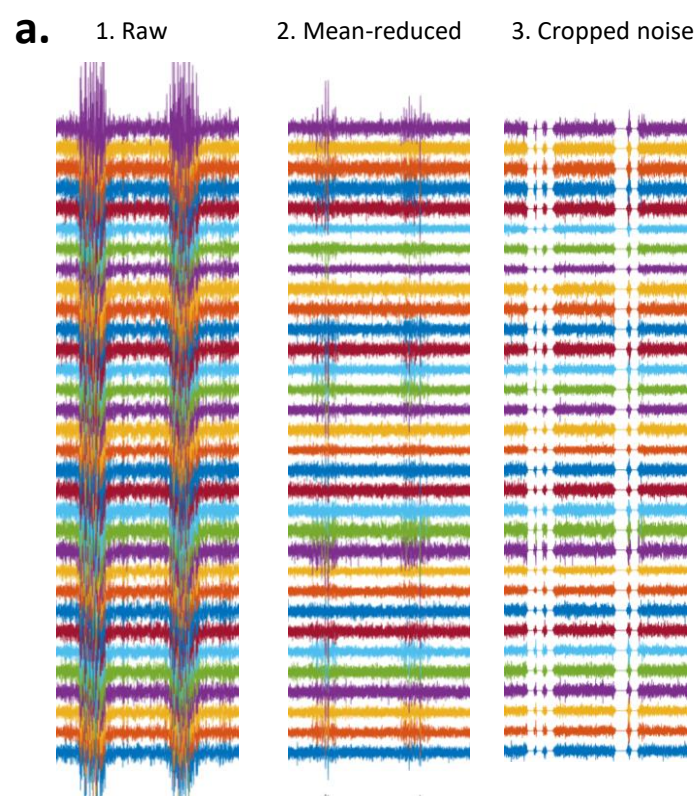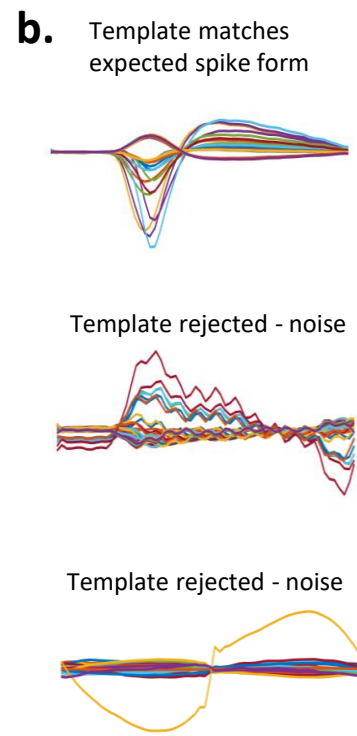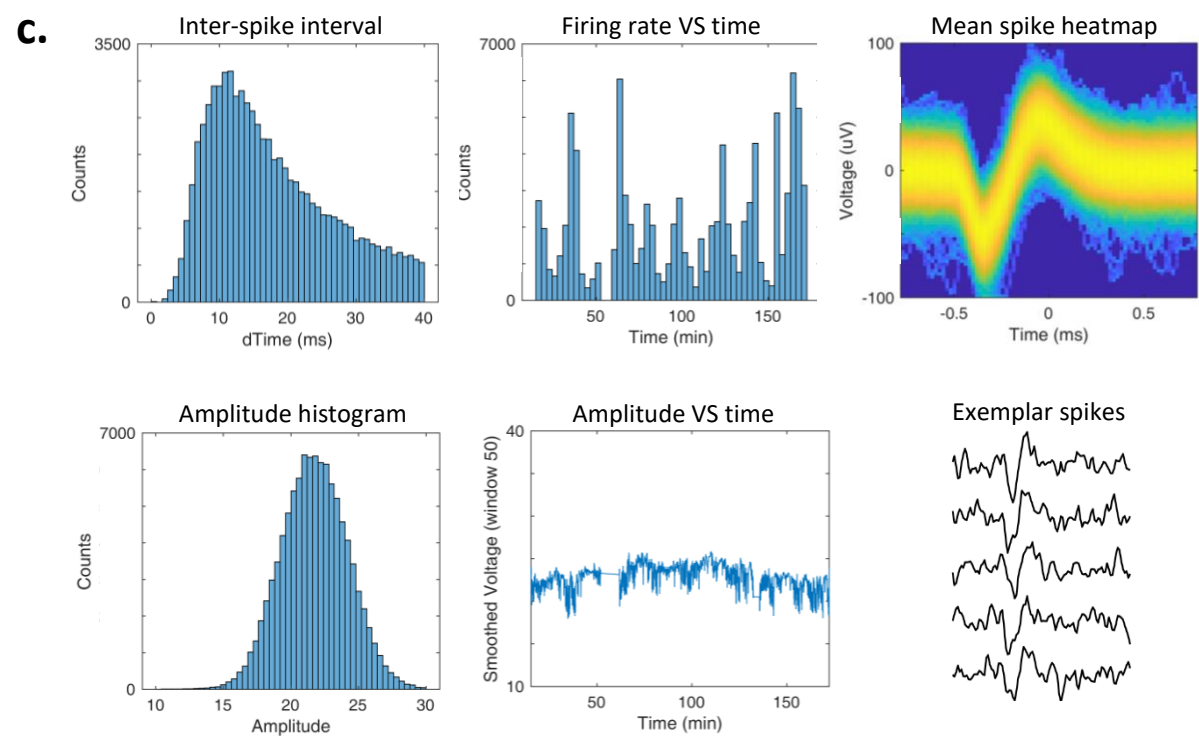

Supplement: Supplementary file 7 — Additional file 7: Figure S5. Neural data processing and spike sorting. (a) Extracellular neural recordings in freely behaving rats (32 simultaneously recorded channels) include periods of high noise (left panel). Noise components that are common to all channels can be largely removed by subtracting the average waveform (middle panel). The remaining noise segments are identified by their amplitude and by their high variance across channels, and are zeroed (right panel). The resulting neural data is then processed by Kilosort2. (b) Implausible spike shapes in the clusters detected by Kilosort2 are automatically detected. The top example was automatically identified as a spike, while the bottom two examples were identified as noise. (c) Statistics of the neural activity are produced for each cluster and used for manual classification. [file 12915_2023_1660_MOESM7_ESM.pdf]

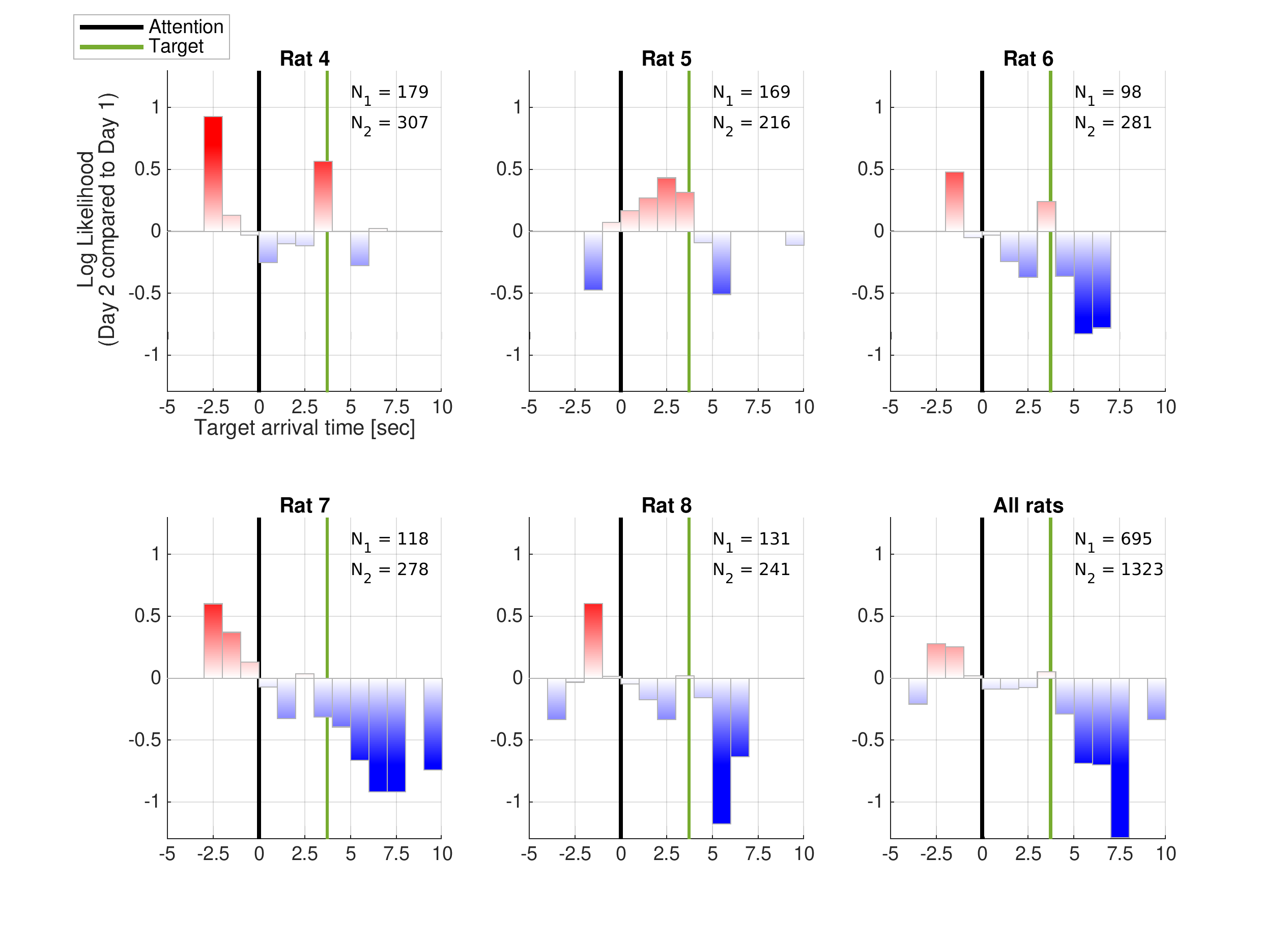

Supplement: Supplementary file 11 — Additional file 11: Figure S6. Changes in target arrival time distributions from Day 1 to Day 2. Shown are log likelihoods of target arrival time in 1 second bins, for Day 2 compared to Day 1. Only rewarded trials are included. Each bar depicts log10 of the probability to arrive at the target in this time bin on Day 2, relative to Day 1. The attention sound is denoted by a black line, and the target sound is denoted by a green line. The number of rewarded trials on each day is denoted in the figure (N1, N2). In each rat, late target arrivals are less likely on Day 2 than on Day 1, and in each rat except rat 5, target arrivals before the attention sound are more likely on Day 2 than on Day 1. The distributions were significantly different in each rat (two-sample Kolmogorov-Smirnov test; P < 0.01 in each rat; P = 4.62 × 10^-15^ for all rats). In all rats except rat 5, target arrival times on Day 2 were significantly earlier than on Day 1 (two-sample t-test; P < 0.006 in rats 4, 6, 7, 8; P = 0.13 in rat 5; P = 1.13 × 10^-26^ for all rats). These data indicate that four out of five rats were more likely to arrive in the target area earlier on Day 2 compared to Day 1 while one rat (rat 5) increased the probability to arrive at the target area between the attention and target sounds relative to earlier and later times. Thus, all rats modified their behavioral strategies in day 2 relative to day 1 in order to better conform to the contingencies of the task. Compare also Fig. 4d in the main text. [file 12915_2023_1660_MOESM11_ESM.png]

**a**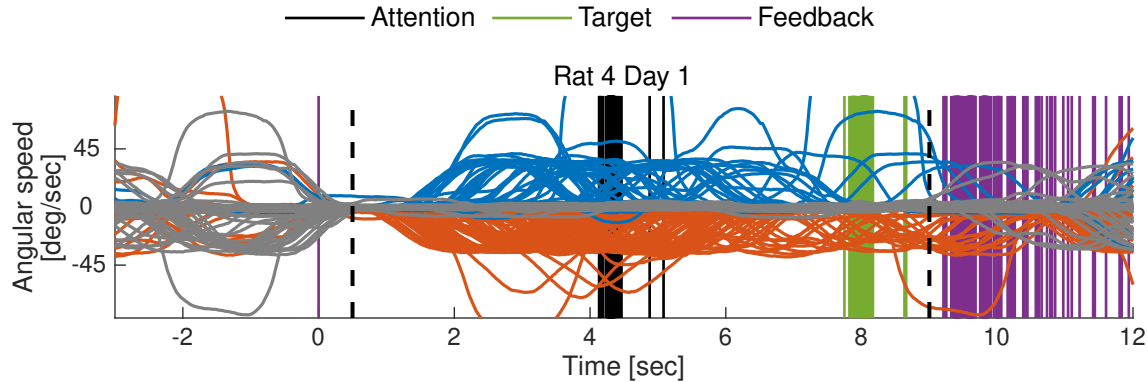**b**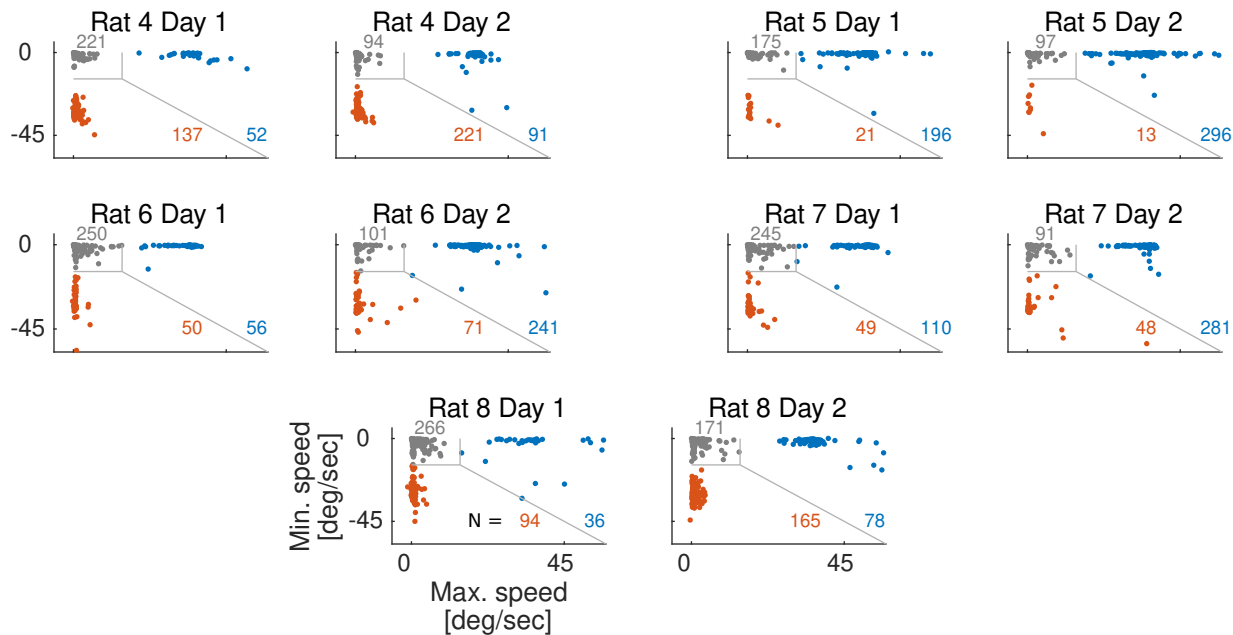

Supplement: Supplementary file 12 — Additional file 12: Figure S7. Classification of trials into three types. (a) Angular running speed for all trials performed by rat 4 on day 1. For each trial, the maximal angular speed in the clockwise and counterclockwise direction was extracted in a time window lasting from 0.5 s to 9 s following the feedback sound of the previous trial. A trial was classified as "Sit" (gray lines) if the absolute value of the angular speed never exceeded 0.25 radians/s (14.3 degrees/s), otherwise as "Run clockwise" (blue lines) or "Run counterclockwise" (red lines), according to the direction with the higher maximal speed. (b) Trial clusters were clearly separated in all rats. Scatter plots show each trial of each rat and each day according to the maximal angular speed in the clockwise and counterclockwise directions. Colors as in (a). The number of trials of each trial cluster are indicated. [file 12915_2023_1660_MOESM12_ESM.pdf]

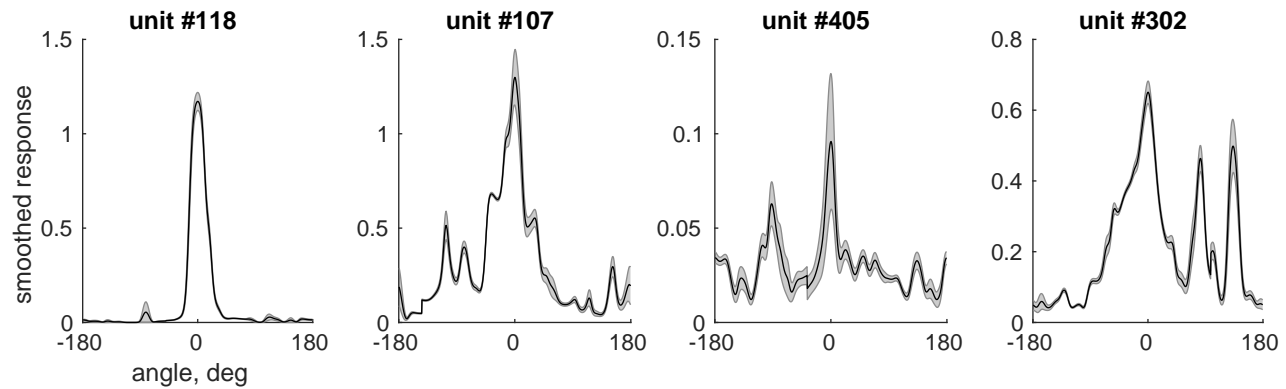

Supplement: Supplementary file 13 — Additional file 13: Figure S8. High-resolution responses of the units in Figs. 5e-h. Response of the units is depicted as probability of firing as a function of the radial location. Each point reflects the weighted average of the firing rates in nearby radial locations. The weights were calculated using a Gaussian window with a standard deviation of 1o. The gray area is standard deviation. In each plot, the maximal response was shifted to 0o. [file 12915_2023_1660_MOESM13_ESM.pdf]

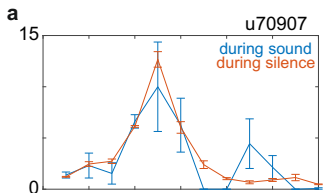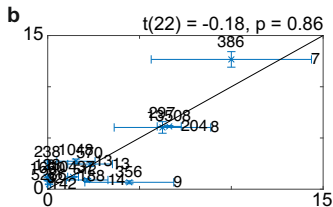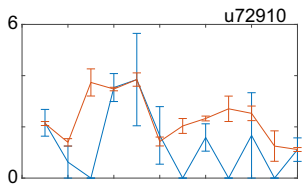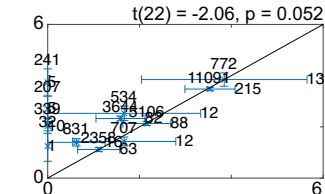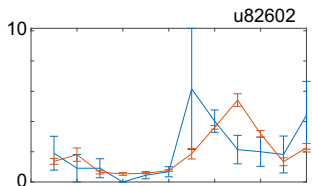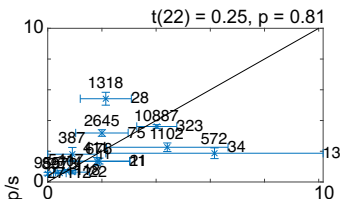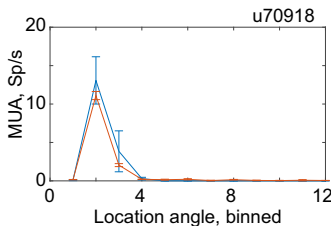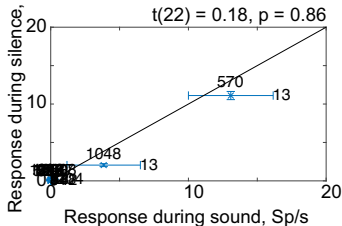

Supplement: Supplementary file 14 — Additional file 14: Figure S9. Joint sensitivity of the units in Figs.5e-h to location and sound. (a) Mean responses in the presence (blue) and absence (red) of sound. Error barsare s.e.m. (b) Scatter plots of the data as in (a). Horizontal error bar indicates s.e.m during sound presentation, vertical error bar indicates s.e.m during silence. The number of instances from which the mean and s.e.m were derived are indicated on the right of each point (during sound presentation), and on top (during silence). In case no sound presentations occurred in a location bin, the data is plotted on the y axis. [file 12915_2023_1660_MOESM14_ESM.pdf]

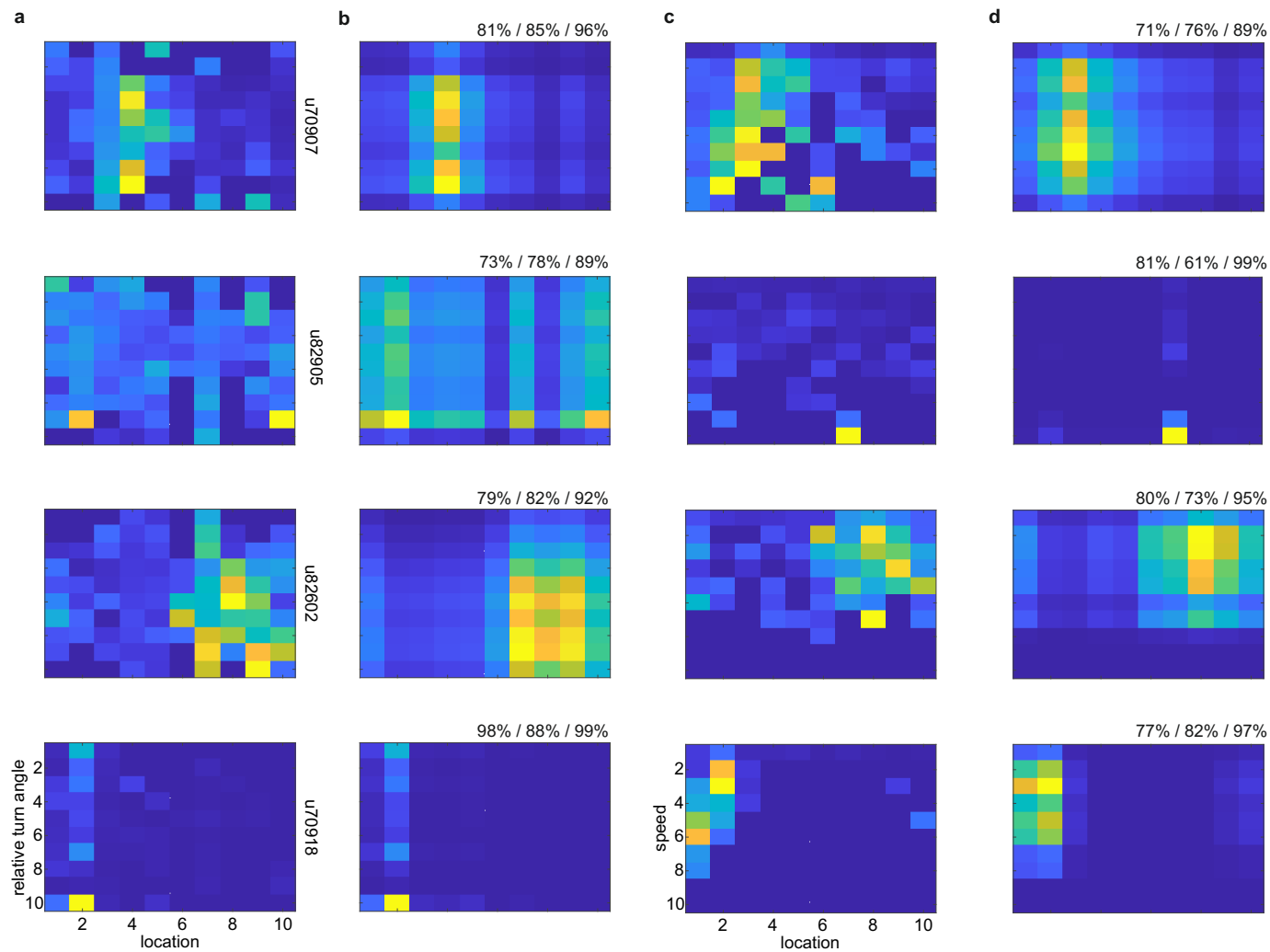

Supplement: Supplementary file 15 — Additional file 15: Figure S10. Joint sensitivity of the units in Figs.5e-h to location and head-body angle or velocity. (a) Mean firing rates for location (abscissa) and relative head-body angle (ordinate). (b) Non-negative rank 1 matrix approximation of the matrices in (a). Top right corner: the fraction of the data variability explained by the rank 1 approximation for the original data / bootstrapping method / Poisson distribution approximation (see "Methods" for details). (c) Mean firing rates for location (abscissa) and velocity (ordinate). (d) Same as (b) for the matrices in (c). [file 12915_2023_1660_MOESM15_ESM.pdf]

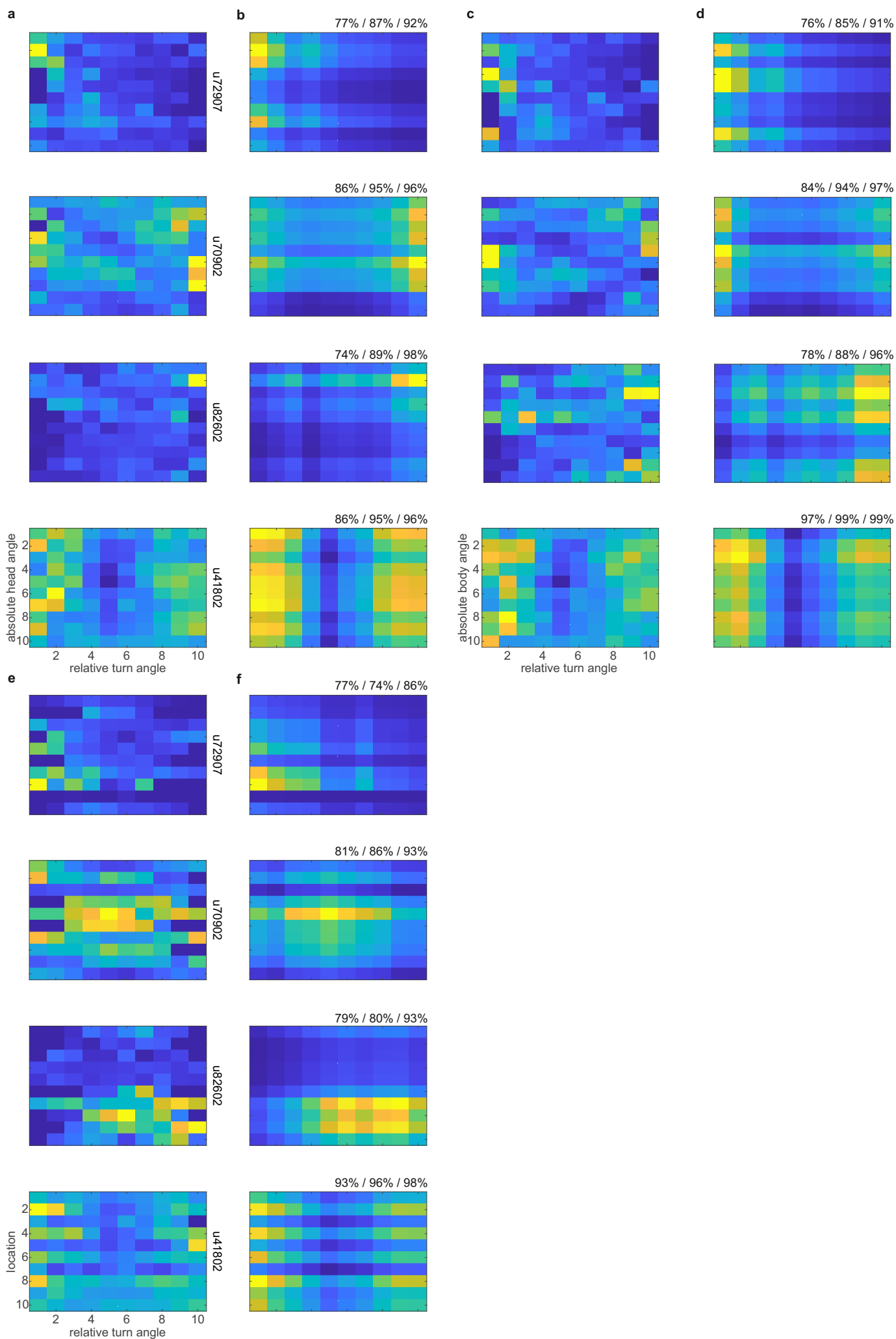

Supplement: Supplementary file 17 — Additional file 17: Figure S12. Joint sensitivity of the units in Figs.5i-l to head-body angle and absolute head angle, absolute body angle, or location. Same representations as in Additional file 15. The abscissa represents head-body angles, while the ordinate represents absolute head angle ((a) and (b)), absolute body angle ((c)and (d)), and location ((e) and (f)). [file 12915_2023_1660_MOESM17_ESM.pdf]

a.

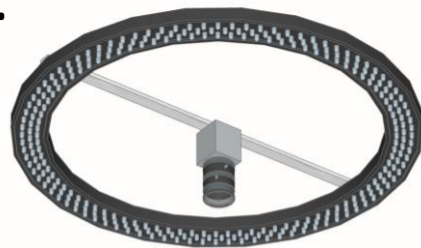

b.

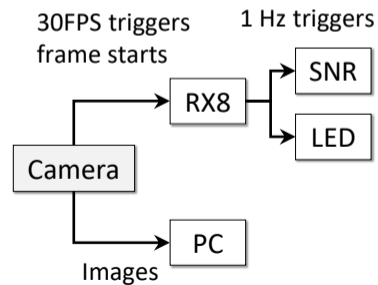

c.

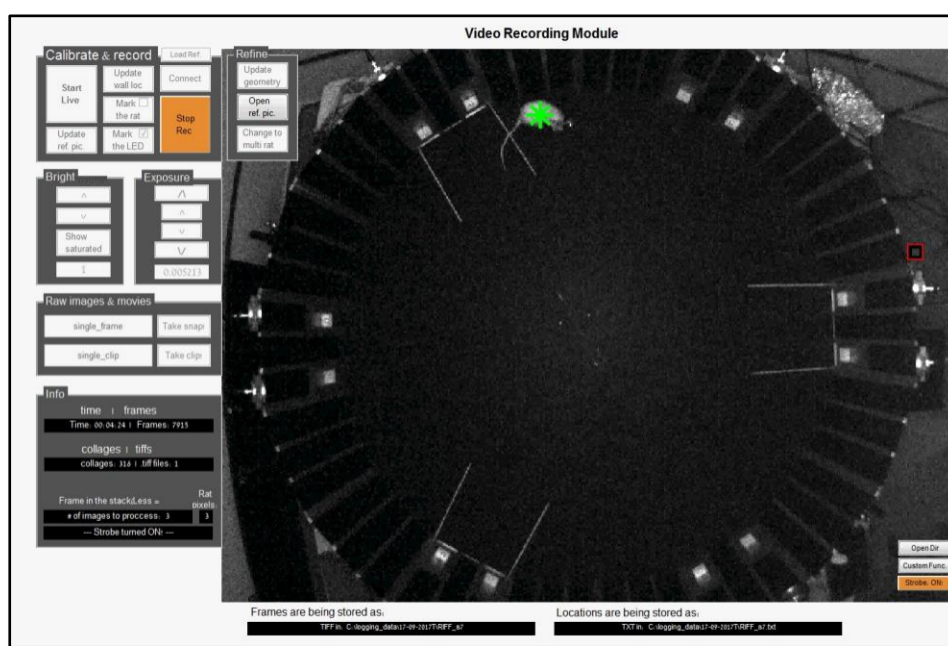

d.

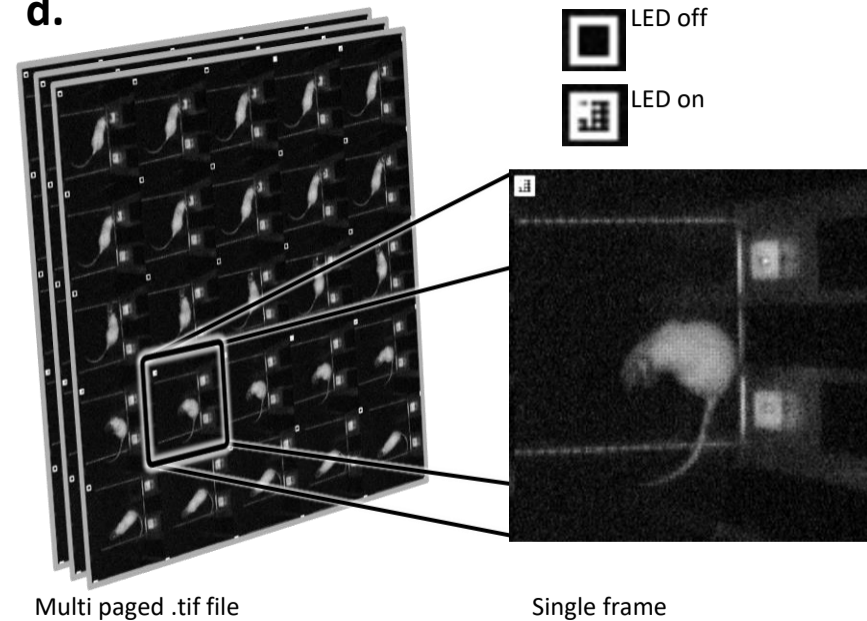

Supplement: Supplementary file 18 — Additional file 18: Figure S13. The real-time imaging module. (a) Diagram of the camera (DMK 33G445 GigE, TheImagingSource) and the light ring, mounted on the ceiling above the arena’s center. (b) Synchronization diagram of the image stream. Triggers that indicate frame acquisition were sent to a digital processor that sub-sampled them from 30 Hz to 1 Hz. The 1 Hz triggers were then simultaneously recorded on the common synchronization hardware, and also powered a LED in the field of view of the camera. (c) Graphical user interface of the real-time imaging module. The LED is marked by a small red square on the right side of the arena. The rat center of mass is marked by a green asterisk. (d) For efficient data storage, rat images were cropped around its center of mass and stored in a multi-page .tif file. A cropped image of the LED was stored in the upper left corner of each image, allowing for time synchronization during the post-processing steps. [file 12915_2023_1660_MOESM18_ESM.pdf]

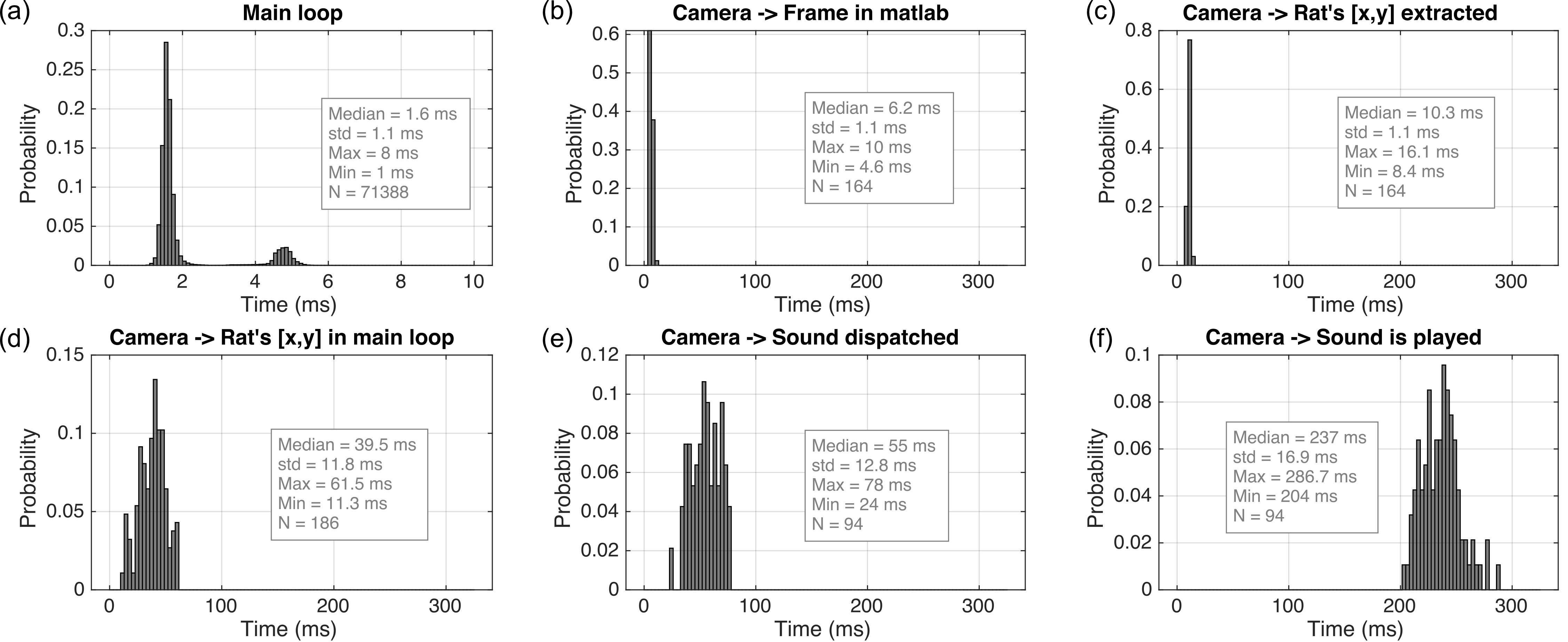

Supplement: Supplementary file 19 — Additional file 19: Figure S14. Analysis of the timing behavior of the main control loop. A. Histogram of running times of the main loop on the master control computer, when no delays were imposed by waiting to external hardware. B-f. Histogram of latencies between image acquisition by the camera and various events on the video processing computer (b and c) and the master control computer (d-f). [file 12915_2023_1660_MOESM19_ESM.png]

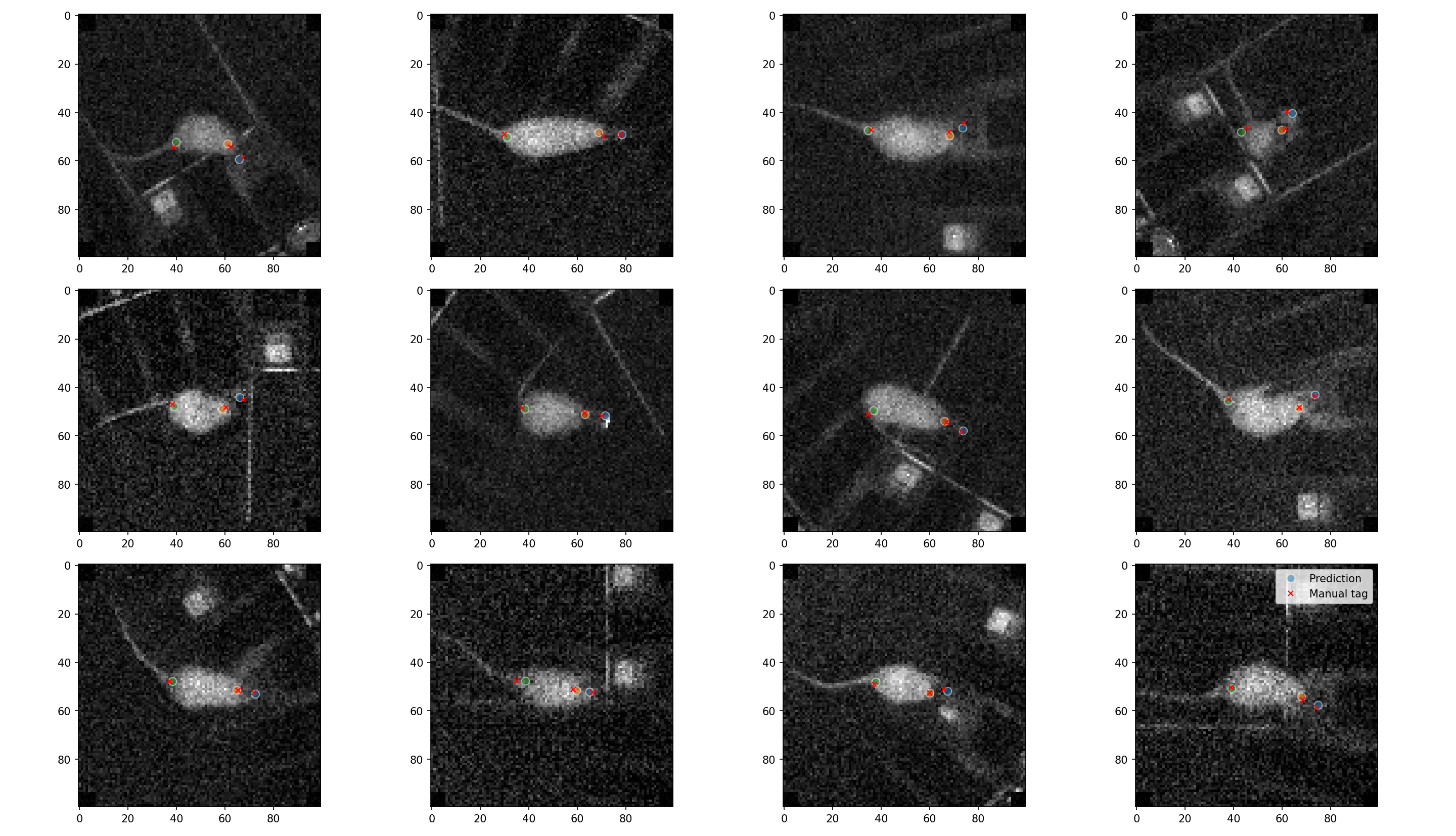

Supplement: Supplementary file 20 — Additional file 20: Figure S15. Exemplary frames of the pose estimation algorithm. The CNN model was trained in a supervised manner to predict the nose, neck and the tail of the rat (blue, orange and green circles, respectively) in 1500 images that were manually tagged (red crosses). [file 12915_2023_1660_MOESM20_ESM.png]

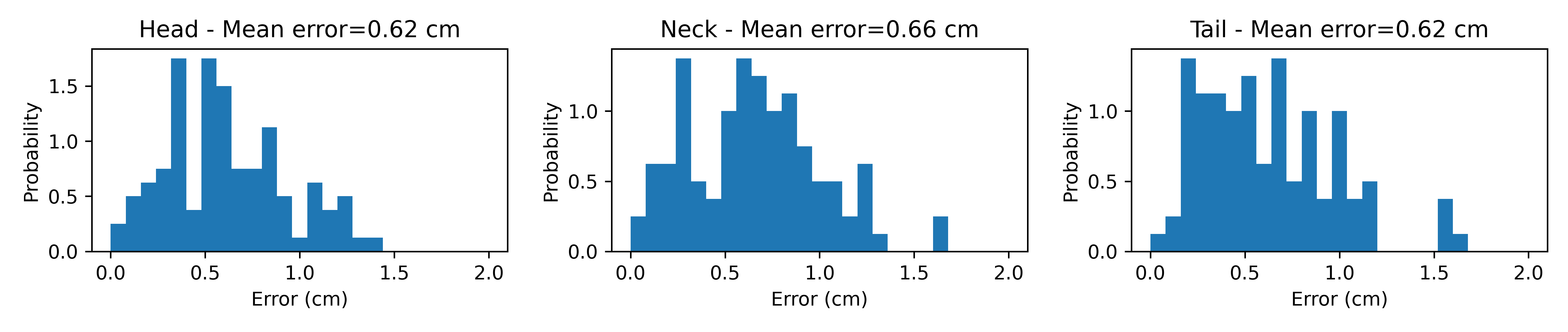

Supplement: Supplementary file 22 — Additional file 22: Figure S16. Accuracy of the pose estimation model. The precision of the model was estimated by calculating the euclidean distance from the predicted points to the manualtags. The mean error for the head, the neck and the tail points was 0.62 cm, 0.66 cm and 0.62 cm, respectively. [file 12915_2023_1660_MOESM22_ESM.png]

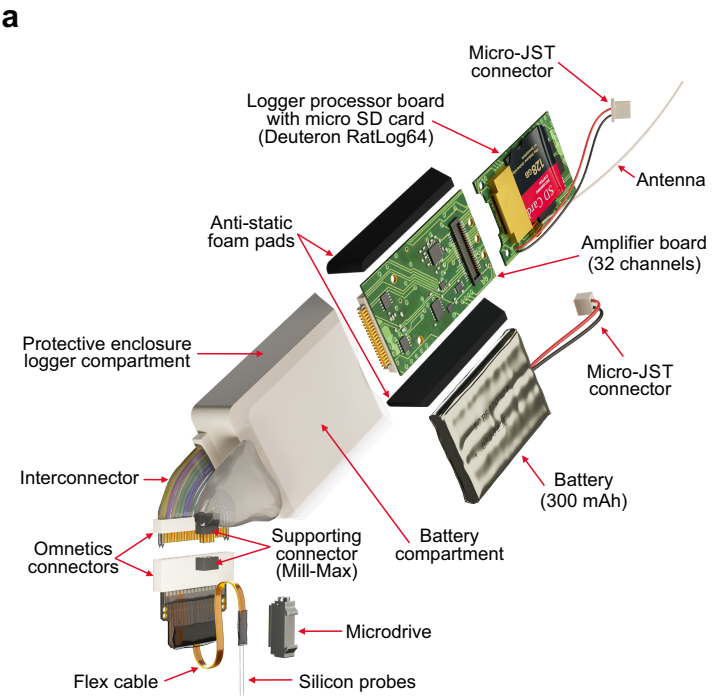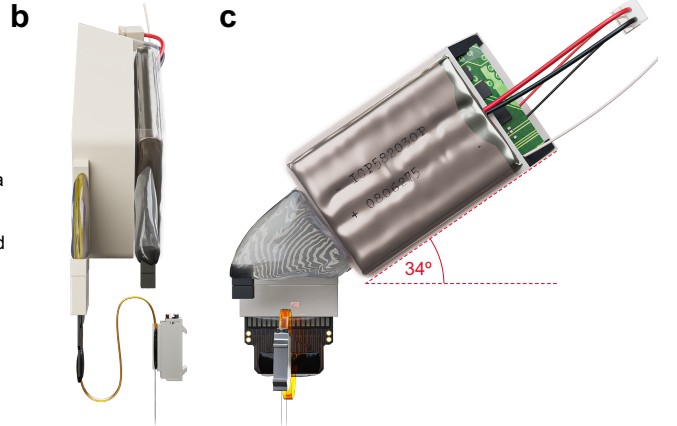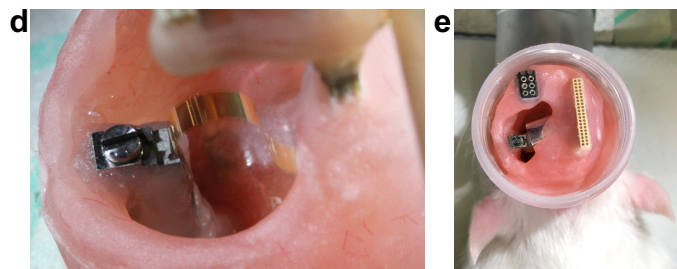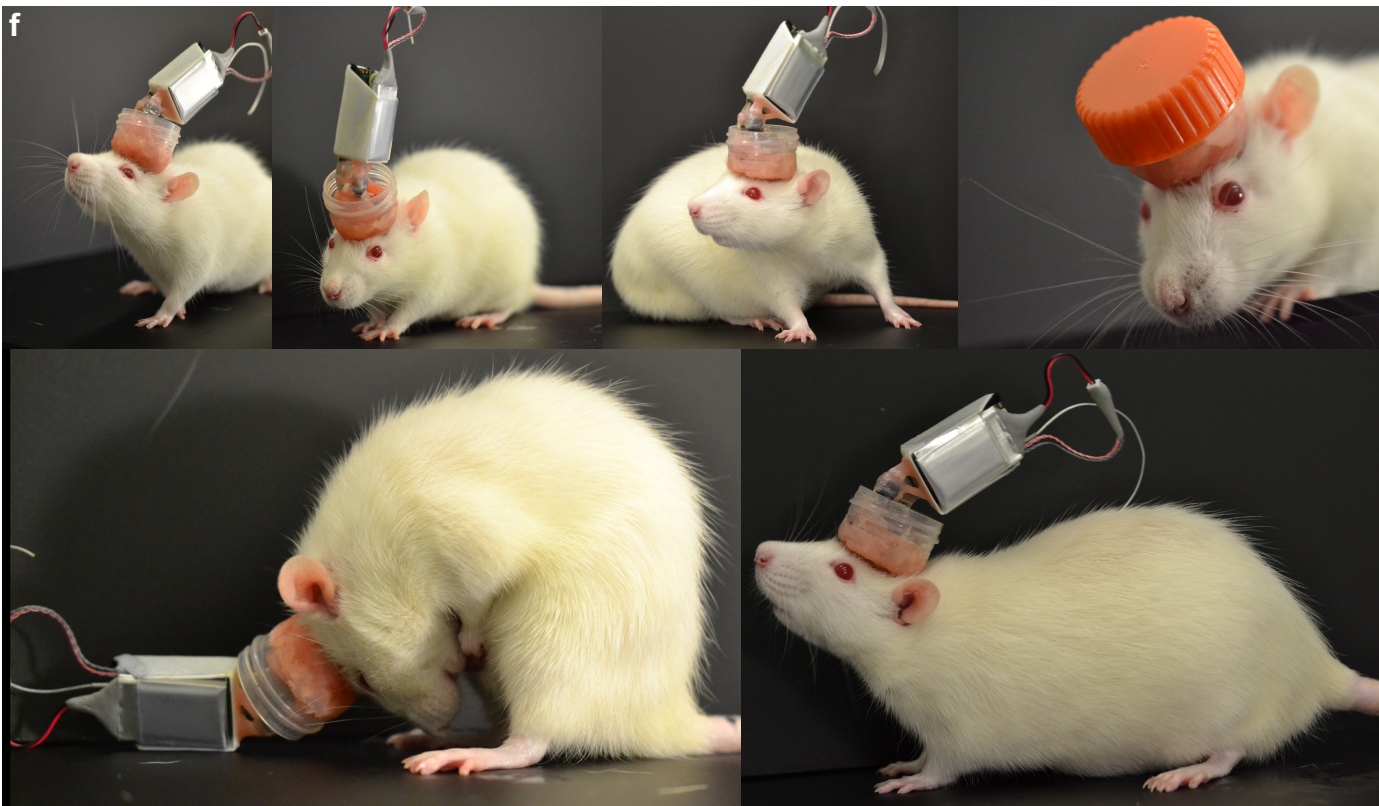

Supplement: Supplementary file 24 — Additional file 24: Figure S17. An approach for chronic wireless recordings in rats. (a) The neural logger and battery are in the protective plastic case that can be attached to the electrode’s connector. The 300 mAh battery is placed in a separate compartment, connects to the logger through a micro-JST connector, and can be easily changed during the experiment. Anti-static foam pads are placed on the sides of the logger components (amplifier and processor boards) to protect the logger against mechanical shocks. The protective case has a 36-pin omnetics connector matching that on the 32-channel silicon probe, as well as a small Mill-Max connector which mechanically stabilizes the case during recording sessions. The silicon probe is mounted on the Microdrive. (b and c) Side and front views of the recording set. The device is inclined to the back in order to allow rats natural movements and undisturbed access to ports. (d) The silicon probe is mounted on the Microdrive cemented to the skull. The moveable parts of the implant are covered with paraffin oil. The flex cable of the probe is bent to provide a long travel distance for the electrodes. (e) Finished implant with protective enclosure. (f) Female rats with a 32-channel moveable silicon probe implant and the wireless data logger in the case with a battery. The recording set enables natural movements, is easily carried by the rats, and is well protected against mechanical shocks. The enclosure can be closed with a plastic cap (orange) to protect the implant in the home cage. [file 12915_2023_1660_MOESM24_ESM.pdf]
