# Supplementary material for: An automated, low-latency environment for studying the neural basis of behavior in freely moving rats
Source: BMC Biol. 2023 Aug 11;21:172. doi: 10.1186/s12915-023-01660-9 (PMC10416379; doi:10.1186/s12915-023-01660-9)
Supplement: Supplementary file 4 — Additional file 4: Figure S4. Feature extraction from the video images. (a) A feed-forward convolutional neural network estimates the locations of the head, the base of the neck and the base of the tail for each input image. These three markers are then used for the calculation of the body and head angles (bottom right image). (b) The table details the custom architecture of the neural network, which is optimized for the grayscale rectangular input, reducing the number of parameters of the trained model and decreasing the inference times. [file 12915_2023_1660_MOESM4_ESM.pdf]

a.

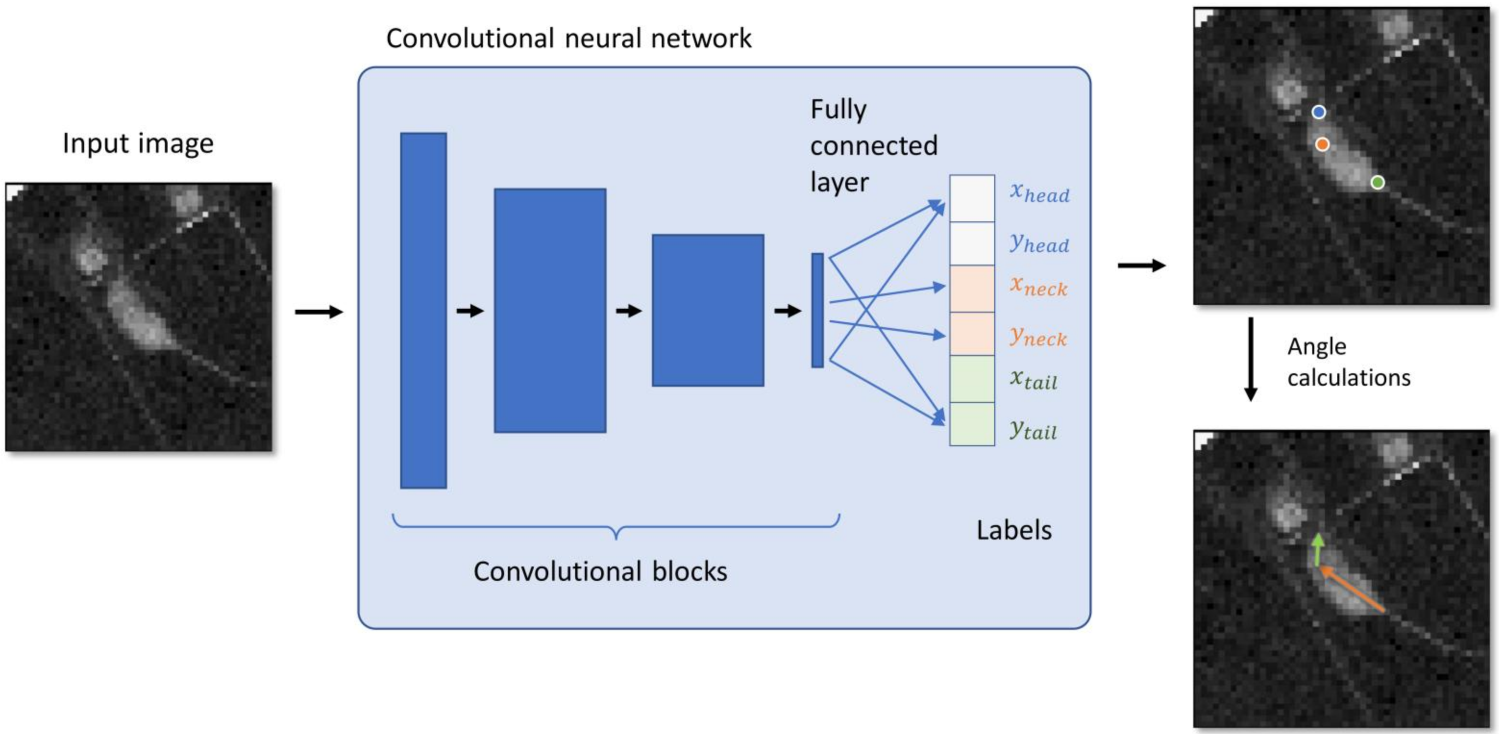

b.

Convolution neural network layers

Type	Input channels	Kernels	Kernel size	Other
Input	1			Input res. 50x50
Convolution	1	32	3	
Batch Norm	32			
ReLU				
Convolution	32	32	3	
Batch Norm	32			
ReLU				
Max Pooling	32		2	Stride = 2
Convolution	32	64		
Batch Norm	32			
ReLU				
Convolution	64	64		
Batch Norm	64			
ReLU				
Max Pooling			2	Stride = 2
Convolution	64	128		
Batch Norm	128			
ReLU				
Convolution	128	128		
Batch Norm	128			
ReLU				
Max Pool	128		2	Stride = 2
Flatten				
Linear	4608	6		
