# Supplementary material for: An automated, low-latency environment for studying the neural basis of behavior in freely moving rats
Source: BMC Biol. 2023 Aug 11;21:172. doi: 10.1186/s12915-023-01660-9 (PMC10416379; doi:10.1186/s12915-023-01660-9)
Supplement: Supplementary file 16 — Additional file 16: Figure S11. Joint sensitivity of the units in Figs.5i-l to location and sound. Same format as Additional file 14. [file 12915_2023_1660_MOESM16_ESM.pdf]

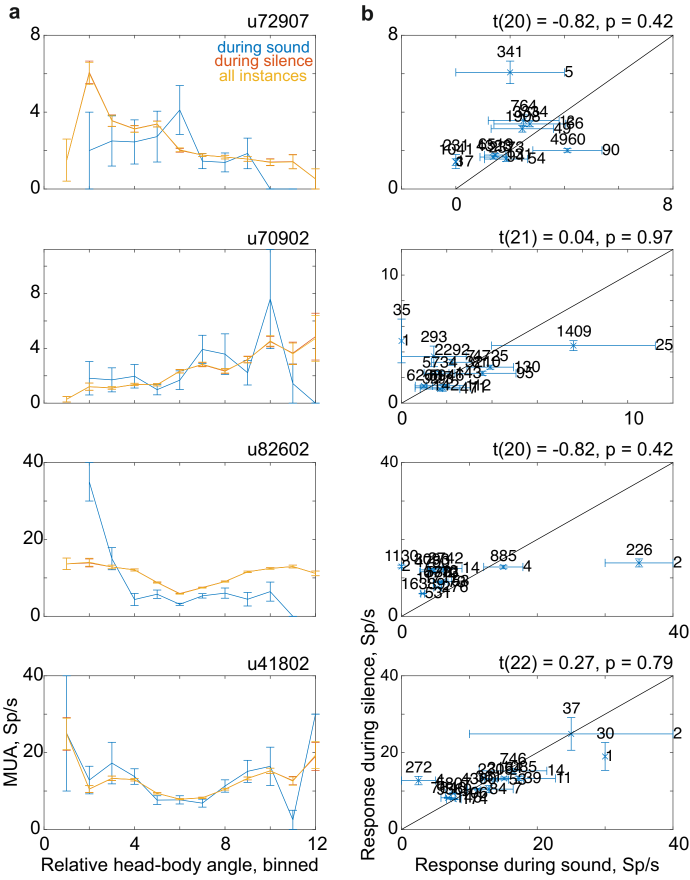
a
b
u72907
during sound
during silence
all instances
t(20) = -0.82, p = 0.42
u70902
t(21) = 0.04, p = 0.97
u82602
t(20) = -0.82, p = 0.42
u41802
t(22) = 0.27, p = 0.79
MUA, Sp/s
Relative head-body angle, binned
Response during silence, Sp/s
Response during sound, Sp/s
